# ClipFaceFusion multi modal diffusion for high fidelity facial generation and modification

**DOI:** 10.1038/s41598-025-31331-4

**Published:** 2025-12-06

**Authors:** Xueming Jiang, Yi Ding

**Affiliations:** 1 College of Art, Suzhou University of Science and Technology, Suzhou, 215011 Jiangsu China; 2 Faculty of Design, Kyiv National University of Technologies and Design, Kyiv, 01011 Ukraine

**Keywords:** Photorealistic face synthesis, Multi-signal conditioning, Diffusion models, Semantic control, Diffusion models, DDIM-based, CLIP-guided synthesis, Cross-modal consistency, Identity preservation, Age and emotion modeling, Audio-visual alignment, Face manipulation, Engineering, Mathematics and computing

## Abstract

The generation of photorealistic human faces utilizing multi-modal inputs presents significant challenges, as existing methodologies like DiffusionCLIP are limited to text-based directives and often struggle with precise attribute control and cross-modal consistency. This paper presents ClipFaceFusion, a diffusion-based framework that amalgamates multi-signal conditioning (text, audio, reference images) with explicit semantic control signals (age and emotion) to produce and alter photorealistic faces. Proposed approach presenting a trainable multi-signal fusion module in conjunction with novel consistency loss functions that provide audio-visual alignment and precise age/emotion regulation within a cohesive Denoising Diffusion Implicit Models (DDIM) framework. Specialized loss functions for age and emotion consistency, along with a multi-tiered identity preservation system utilizing ArcFace, perceptual loss, and reference image alignment, ensure precise attribute regulation and identity conservation. Experiments demonstrate that ClipFaceFusion outperforms leading techniques such as DiffusionCLIP and StyleCLIP in generating realistic faces with precise age and emotional expressions, while facilitating dependable image modification. The framework facilitates applications in media creation, psychological simulations, historical facial reconstruction, and interactive virtual environments by providing superior Cross-Modal Coherence (CMC) and reduced visual artifacts. ClipFaceFusion seamlessly integrates multi-modal inputs into a unified diffusion-based model, establishing a new benchmark for personalized face synthesis and manipulation.

## Introduction

The generation of photorealistic human faces has emerged as a significant challenge in computer vision, owing to its applications in media creation, virtual reality, psychology, and historical reconstruction^1^. Recent advancements in generative models, particularly Generative Adversarial Networks (GANs) and diffusion-based models, have significantly enhanced the quality of synthetic faces. GAN-based methodologies, shown as StyleGAN^2^, have achieved remarkable photorealism^3^. However, they often have difficulties in precise attribute modification and maintaining consistency across several domains. Diffusion models, including Denoising Diffusion Probabilistic Models (DDPM)^4^ and Denoising Diffusion Implicit Models (DDIM), provide improved stability and fidelity through the iterative denoising of random noise to generate high-quality images^5^. The utilization of pre-trained vision-language models like CLIP^6^ has revolutionized text-guided picture synthesis by facilitating the intuitive manipulation of visual features using natural language, as illustrated in DiffusionCLIP. Nonetheless, current methodologies mostly depend on single-modal inputs, usually text or photos, constraining their capacity to encompass the complex, multifaceted aspects of human facial traits, including emotional expressions, age-related features, audio-derived signals, and identity preservation based on reference images.

Photorealistic facial synthesis, directed by multi-modal inputs, possesses significant potential for applications necessitating individualized and contextually consistent results. In media production, creating faces with distinct ages and emotions enriches character design and narrative development^7^. In psychology research, the simulation of emotional states facilitates investigations into human behavior and affective computing^8^. Historical reconstruction is enhanced by age and emotion accurate facial representations, but virtual reality necessitates dynamic, high-fidelity faces that correspond with auditory and emotional signals^9^. The integration of varied inputs textual descriptions, auditory signals, age parameters, emotional states, and reference images into a cohesive framework is poised to transform generative modeling, providing a multifaceted tool for the creation and manipulation of highly personalized, realistic faces.

In addition to face synthesis, multimodal learning has been thoroughly investigated across several vision tasks, showcasing its efficacy in using diverse inputs. Previous research in pose estimation and human analysis, including multimodal manifold learning for face-pose prediction^10^, deep autoencoder-based multimodal pose recovery^11^, and multi-view sparse retrieval for 3D pose reconstruction^12^, collectively demonstrates that complementary modalities can enhance robustness and structural consistency. While these efforts pursue distinct goals, they underscore the overarching significance of multimodal fusion frameworks and emphasize the necessity for more sophisticated models that can amalgamate varied information sources. Our research primarily addresses the multimodal synthesis and modification of photorealistic human faces, necessitating the concurrent alignment of text, voice, age, emotion, and reference identification within a diffusion-based generative model.

Notwithstanding these gains, numerous obstacles remain in multi-modal face generation and manipulation. Attaining CMC across varied inputs (e.g., text, audio, age, mood, and reference images) is complex, as modalities operate within distinct feature spaces defined by varying levels of abstraction. Though they have greatly enhanced photorealism, attribute control, and cross-modal understanding, recent developments in facial representation learning^13^, expressive facial animation via latent diffusion^14^, text-driven facial attribute editing^15^, multimodal generative AI^16^, and audio-visual emotion recognition^17^ are either restricted to single-modality conditioning or lack precise simultaneous control over age, emotion, audio-driven expression, and identity preservation in a unified diffusion framework. The creation and alteration of photorealistic faces with precise age, emotional characteristics, and identity retention require meticulous management of intricate details, including skin texture and nuanced expressions, which existing models do not reliably accomplish across diverse datasets like FFHQ^2^, VoxCeleb^18^, or CACD^19^.

This study addresses the constraints of single-modal face synthesis by utilizing several input modalities for photorealistic face generation and modification. Although text-guided techniques such as DiffusionCLIP^14^ provide intuitive control through natural language and reference images, they do not adequately encompass the complexity of auditory signals (e.g., tone, emotional inflection) or the accuracy of age and emotional parameters^20^. Audio signals convey implicit information on gender, age, or emotional states, so augmenting the realism and personalization of created or altered faces^21^. Explicit inputs of age and mood, along with reference photos, provide accurate manipulation of facial attributes required for applications such as historical restoration or psychological simulation^22^. By consolidating these modalities into a cohesive framework, we intend to develop a generative model that generates high-fidelity faces and modifies existing images while effortlessly accommodating various input situations, thereby addressing the limitations of current methodologies.

**Fig. 1:**
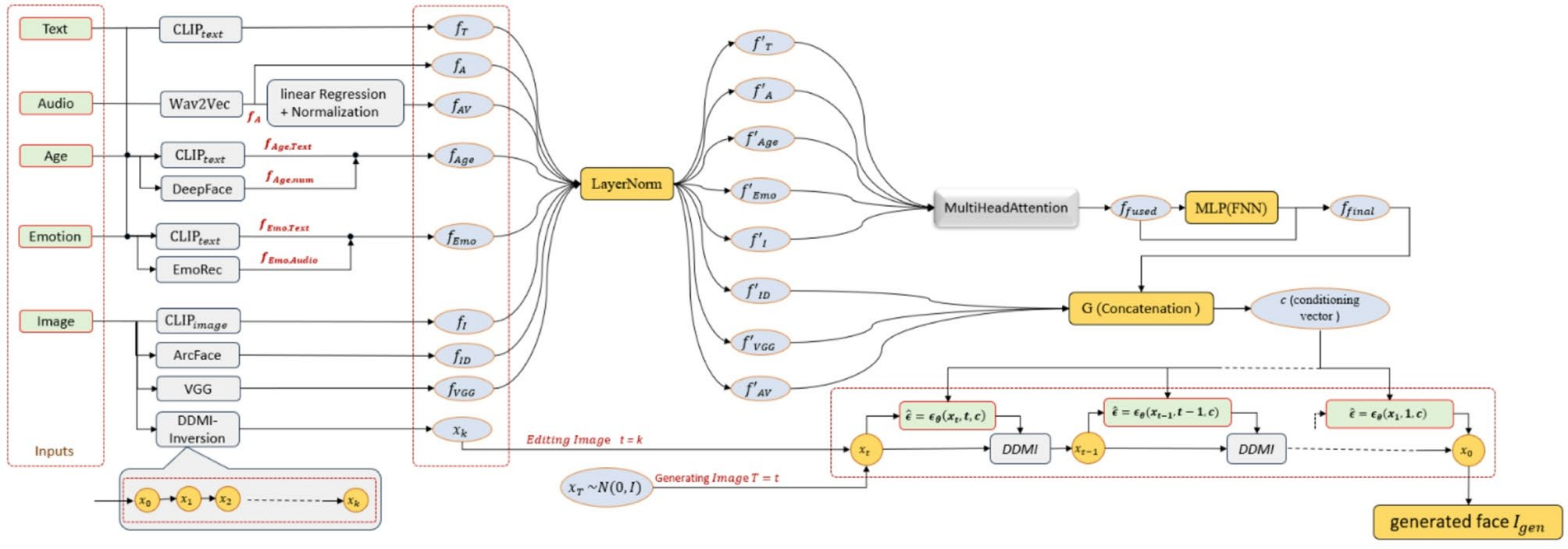
Text prompts, audio waveforms, explicit age and emotion criteria, and reference images are initially encoded into latent feature vectors utilizing pre-trained encoders. A multi-head attention-based fusion module integrates signal modalities and semantic control signals to generate a cohesive conditioning vector. This vector, in conjunction with identification traits derived from the reference image, directs the DDIM-based reverse diffusion process to produce or modify a photorealistic face.

A crucial conceptual differentiation is necessary: signal modalities refer to separate sensory input streams (e.g., text, audio, and visual imagery), each represented inside fundamentally different feature spaces. In contrast, semantic attributes such as age and emotion serve as high-level descriptors that can be communicated through one or several signal modalities or provided explicitly as user-defined control parameters. ClipFaceFusion identifies text, audio, and reference images as the principal signal modalities, while age and mood are regarded as semantic control signals. Control signals can be obtained from: textual descriptions (e.g., “a 25-year-old happy woman”), numerical specifications (e.g., age = 25), audio-derived inferences (e.g., pitch indicating perceived age, prosody communicating emotional state), or direct parameter input. This stringent separation enables strong cross-modal alignment of semantic features while reducing the frequent mistake of merging high-level annotations with distinct sensory channels. The suggested taxonomy is explicitly delineated in “Framework synopsis” and depicted on Fig. 1.

This study presents ClipFaceFusion, an innovative framework that amalgamates textual descriptions, audio signals, age characteristics, emotional states, and reference photos into a diffusion-based model for photorealistic facial synthesis and modification. In contrast to DiffusionCLIP^14^, which depends exclusively on text and a reference image, ClipFaceFusion utilizes a multi-modal fusion module to integrate various inputs, hence assuring CMC. An audio-visual alignment module associates aural features with visual properties, while specific age and emotion consistency losses, combined with reference image-based multi-tiered identity preservation (ArcFace^23^, perceptual loss, and alignment of reference images), ensure meticulous control over facial characteristics. Utilizing DDIM^13^ and CLIP^6^, our methodology attains resilient latent optimization and enhanced synthesis quality, setting it apart from GAN-based and single-modal diffusion techniques.

This research contributes to the domain of photorealistic facial synthesis in the following ways:

- Multi-Modal Integration: We present a framework that amalgamates text, audio, age, emotion, and reference images, facilitating tailored facial synthesis with exact attribute regulation.
- Audio-Visual Alignment: A specialized module for audio-visual alignment combines auditory information (e.g., tone, emotional inflection) with visual attributes, augmenting the realism and expressiveness of synthesized and modified faces.
- Targeted Consistency Losses: We present age and emotion consistency losses, integrated with a multi-tiered identity preservation framework (ArcFace^23^, perceptual loss, and reference image alignment), to guarantee precise attribute regulation and identity maintenance.
- Reference Image Integration: ClipFaceFusion facilitates zero-shot image editing by the incorporation of reference images, maintaining identity and enhancing its multi-modal synthesis capabilities, hence surpassing text-only methodologies such as DiffusionCLIP^14^.
- Exceptional Performance: Experimental findings indicate that ClipFaceFusion outperforms leading techniques, like DiffusionCLIP and StyleCLIP^24^, in the generation and manipulation of photorealistic faces with accurate age, emotional expressions, and cross-modal consistency.

Previous research utilizes pre-trained encoders (e.g., CLIP, Wav2Vec), but ClipFaceFusion is distinguished by three interrelated technical innovations:

- A unified multi-signal and semantic-control conditioning facilitated by a trainable fusion module that concurrently enforces cross-modal alignment and attribute consistency.
- Task-oriented consistency losses (Audio-Visual, Age, Emotion) that directly limit latent drift during DDIM inversion and the reverse sampling phase;
- Identity preservation based on reference via multi-tier constraint mechanisms (ArcFace + perceptual + alignment) that provide zero-shot, identity-consistent modifications across age and emotion.

These contributions are not merely combinatorial; they establish novel optimization objectives and architectural limitations absent in previous fusion-based systems. In summary, ClipFaceFusion presents a cohesive multi-signal diffusion framework featuring innovative alignment and consistency objectives, establishing it as the inaugural model to concurrently incorporate audio-driven expression synthesis, explicit age modulation, emotion regulation, and identity-preserving zero-shot editing.

The subsequent sections of this work are structured as follows. Section “Related work” examines pertinent literature on diffusion models, text-guided synthesis, and multi-modal generative methodologies. Section “The clipfacefusion framework” delineates the proposed ClipFaceFusion framework, encompassing its architecture, multi-modal fusion, and optimization objectives. Section “Experiments” delineates experimental outcomes, encompassing datasets, evaluation metrics, and comparisons with leading methodologies. Section “Conclusion” delineates the benefits, constraints, and utilizations of our methodology, culminating in principal discoveries and prospective research trajectories.

## Related work

This section examines current progress in facial synthesis, modification, and detection, emphasizing GANs, diffusion models, multi-modal strategies, and CLIP-guided techniques. These works establish the basis for ClipFaceFusion, which amalgamates multi-modal inputs text, audio, age, emotion, and reference images into a cohesive diffusion-based framework for photorealistic face generation and manipulation.

GANs have proved essential in the synthesis of photorealistic faces. StyleGAN^2^ attains remarkable realism through style-based generation, facilitating the modulation of variables such as age and mood via latent space adjustments. Nonetheless, GANs frequently encounter difficulties in accurate attribute modification and identity retention in intricate situations^3^. Techniques such as PTI^25^ and HyperStyle^26^ improve GAN inversion for the reconstruction of real images, enabling the modification of latent codes for various properties. GANSpace^16^ and SeFa^27^ investigate latent vectors for semantic modifications, including alterations in stance or expression. StyleCLIP integrates CLIP embeddings with StyleGAN for text-directed modifications, facilitating alterations such as “happy face” or “elderly man.” Notwithstanding these advancements, GAN-based methodologies are plagued by mode collapse, artifacts in out-of-distribution poses, and restricted multi-modal integration, necessitating a transition to diffusion models for enhanced stability and diversity in generation.

Diffusion Models (DMs) have eclipsed GANs in image production, providing robust training and high-fidelity results^5^. DDPM^4^ and DDIM^13^ provide the essential denoising framework for iterative enhancement. Recent evaluations underscore the effectiveness of DMs in augmenting picture data^28^. Stable Diffusion^29^ facilitates text-conditioned generation, whereas GODiff^30^ employs CLIP-guided diffusion models for region-specific semantic editing, emphasizing precise alterations without impacting extraneous areas, which is advantageous for facial editing applications. DiffusionAct^31^ employs tunable diffusion autoencoders for one-shot face reenactment, maintaining identity while conveying expressions. RigFace^32^ integrates 3D morphable models with deep models for coherent facial editing, regulating illumination, posture, and expression. These DM-based systems excel in producing diverse, realistic images. nevertheless, they frequently exhibit deficiencies in multi-modal integration beyond text, constraining their utility in audio-driven or age-specific facial synthesis.

Multi-modal facial generation integrates several inputs for improved control. CLIP-Forge^33^ integrates text-to-shape generation through CLIP embeddings with diffusion priors, facilitating zero-shot 3D shape synthesis from textual descriptions. BrainCLIP^34^ enhances CLIP by interpreting visual stimuli from fMRI signals, merging cerebral activity with image-text domains for stimulus reconstruction. SynAdult^35^ produces synthetic adult datasets utilizing deep models and neuromorphic simulation for biometric applications, highlighting privacy and multi-modality in age-specific data. MFCLIP^36^ utilizes multi-modal fine-grained CLIP for the detection of face forgeries, integrating image-noise characteristics with text for cross-modal alignment. These studies illustrate CLIP’s adaptability in multi-modal tasks; nevertheless, few focus on face-specific synthesis utilizing integrated text, audio, age, mood, and reference image inputs. ClipFaceFusion enhances this by integrating various modalities within a DDIM framework, guaranteeing accurate attribute control and identity retention.

Deepfake technologies engender ethical dilemmas regarding facial alteration. Reviews emphasize GANs and DMs for Deepfake production and identification, indicating DMs’ superiority in generating hyper-realistic faces^37^. One-shot Face Sketch Synthesis^38^ employs generative diffusion priors to generate sketches from genuine photos, facilitating counterfeit detection. These findings highlight the necessity for effective detection in multi-modal environments, which ClipFaceFusion mitigates through its identity preservation approach, hence diminishing susceptibility to Deepfake-like effects.

HydraMamba^39^ presents a multi-head state space model for global point cloud learning, enhancing selective state space models (S6) to address long-range dependencies in 3D data. Although largely applicable to point clouds, its ideas also inform multi-modal fusion in facial synthesis. ClipFaceFusion enhances these models by integrating multi-head attention in the fusion process, attaining comparable long-range coherence specifically for 2D face generation.

In conclusion, whereas GANs are proficient in style manipulation and CLIP-guided techniques facilitate text-based modifications, DMs provide reliability for high-quality generation. ClipFaceFusion uniquely amalgamates multi-modal inputs with reference photos within a DDIM pipeline, rectifying deficiencies in attribute accuracy and identity retention.

Recent advancements in multi-modal diffusion have unveiled progressively adaptable conditioning techniques. VersatileDiffusion^40^ integrates text, edge maps, and depth via modular adapters; nevertheless, it does not include audio processing and lacks fine-grained attribute management. MM-Diffusion^41^ integrates text and image embeddings through cross-attention; nonetheless, it lacks support for specific age or emotion criteria and does not facilitate audio-driven expression development. EmoDiff^42^ employs classifier-free guiding utilizing emotion labels; nevertheless, its conditioning is only focused on text-based emotion tokens, neglecting audio prosody. AudioFace^43^ converts speech into 3D facial motion, operating within a parametric FLAME space without generating photorealistic 2D images. DiffFace^44^ executes identity-preserving modifications via inversion, although it is constrained to text and reference image conditioning.

Conversely, ClipFaceFusion represents the fundamental framework to: (1) Integrate audio as a principal control medium with adaptable audio-visual alignment. (2) Supply specific age and emotional inputs (either numeric or categorical) reinforced by specialized consistency losses. (3) Attain zero-shot, multi-attribute manipulation while maintaining robust identity preservation inside a cohesive DDIM framework. The distinctions are encapsulated in Table 1.

**Table 1:** Technical comparison with current multi-modal diffusion models.

Method	Audio input	Age control	Emotion control	AV-AL	AE-CL	IP-MT	Zero-shot edit
VersatileDiffusion^40^	✗	✗	✗	✗	✗	✗	✓
MM-Diffusion^41^	✗	✗	✓*	✗	✗	✗	✓
EmoDiff^42^	✗	✗	✓	✗	✗	✗	✓
AudioFace^43^	✓	✗	✓*	✗	✗	✗	✗
DiffFace^44^	✗	✗	✗	✗	✗	✓	✓
ClipFaceFusion (proposed)	✓	✓	✓	✓	✓	✓	✓

AV-AL: Audio-Visual Alignment Loss; AE-CL: Age/Emotion Consistency Loss; IP-MT: Multi-Tiered Identity Preservation; ✓*: Limited support.

To clarify the advancements of ClipFaceFusion, we juxtapose it with prominent techniques in face synthesis and modification, encompassing GAN-based, diffusion-based, and multi-modal methodologies. Table 2 delineates the comparison of input modalities, core architecture, major capabilities, and limits, emphasizing ClipFaceFusion’s distinctive amalgamation of text, audio, age, emotion, and reference images inside a DDIM-based framework for photorealistic face synthesis.

**Table 2:** A comparative analysis of clipfacefusion against principal methodologies in facial generation and modification, assessed by input modalities, foundational architecture, functionalities, and constraints.

Method	Input modalities	Core architecture	Primary capabilities	Limitations
StyleGAN^2^	Noise vector	Style-based GAN, AdaIN modulation	High-fidelity face synthesis, style control	Limited attribute precision, mode collapse, no multi-modal input support
StyleCLIP^24^	Image, text prompt	CLIP + StyleGAN, latent optimization	Text-guided semantic editing (e.g., “happy face”)	Artifacts in complex poses, limited to text-image inputs
DiffusionCLIP^14^	Image, text prompt	CLIP-guided DDIM	Robust text-driven manipulation, zero-shot editing	Text-only guidance, struggles with emotional and age precision
GODiff^30^	Image, text prompt	CLIP-guided DM, region-specific editing	Precise semantic editing, minimal irrelevant changes	Lacks audio/emotion integration, limited age control
MFCLIP^36^	Image, text, noise features	Multi-modal CLIP, fine-grained noise encoder	Face forgery detection, cross-modal alignment	Focused on detection, not synthesis; limited age/emotion control
SynAdult^35^	Text, age-specific prompts	DMs, neuromorphic simulation	Synthetic adult dataset generation, privacy-preserving	Limited to biometric applications, no audio/emotion integration
HydraMamba^39^	Point cloud data	Multi-head state space model (S6)	Long-range dependency modeling for 3D data	Primarily for 3D point clouds, not tailored for 2D face synthesis
ClipFaceFusion (proposed)	Text, audio, age, emotion, reference image	DDIM, multi-modal fusion, audio-visual alignment	Photorealistic synthesis, precise age/emotion control, identity preservation, zero-shot editing	High computational cost, dependency on high-quality reference images

## The clipfacefusion framework

This section introduces ClipFaceFusion, a comprehensive diffusion-based framework for generating and altering photorealistic faces utilizing various input signals, including written descriptions, audio cues, explicit age and emotion parameters, and reference photos. In contrast to previous diffusion or CLIP-guided models, ClipFaceFusion amalgamates diverse inputs via a learnable multi-modal fusion module, ensures consistency across audio-visual elements, age, and emotion, and maintains identity through a multi-tier reference-guided mechanism (ArcFace combined with perceptual constraints). Figure 1 presents a comprehensive overview of the system, demonstrating the encoding, fusion, and application of each input stream to condition the DDIM sampling process.

### Framework synopsis

**Fig. 2:**
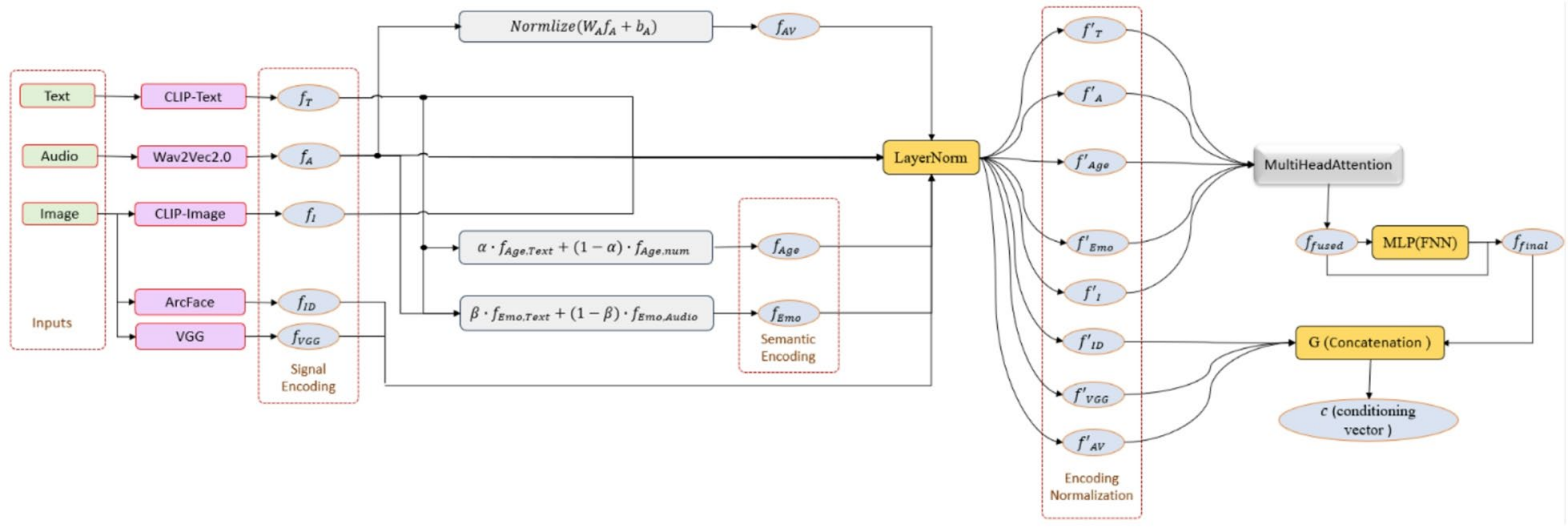
Illustrates the system architecture and multi-modal data flow in ClipFaceFusion, summarizing the processing of text, audio, age, emotion, and reference-image inputs, along with their integration via the fusion module prior to DDIM-based production.

ClipFaceFusion is engineered to produce and modify photorealistic human faces by amalgamating various input signals textual descriptions, auditory cues, explicit age and emotional parameters, and reference images within a cohesive DDIM-based diffusion framework. The framework functions in four steps, as depicted in Fig. 2.

- Input Encoding: Each input stream is initially transformed into a concise latent representation utilizing pre-trained encoders (CLIP for text and graphics, Wav2Vec 2.0 for audio, and age/emotion estimators for semantic control signals).
- Multi-Modal Fusion: The encoded characteristics are normalized and integrated by a learnable multi-head attention module, resulting in a singular, cohesive conditioning vector that encapsulates both signal modalities and semantic control properties.
- Constraint Modules: Specialized modules ensure synchronization between auditory and visual signals (Audio-Visual Alignment), maintain consistency in age and emotional expression, and uphold identity relative to the reference image through ArcFace- and perceptual-based restrictions.
- Diffusion-Based Generation: The consolidated conditioning vector and constraint signals direct a DDIM sampler that originates from partially noisy reference latents (for editing) or pure noise (for synthesis) and progressively generates a high-fidelity facial image that adheres to all inputs.

### Input taxonomy and encoding

#### Signal modalities

This section delineates the methodology by which ClipFaceFusion models the various input streams presented in “Framework synopsis”. This method differentiates between signal modalities (text, audio, and reference image) and semantic control signals (age and emotion), detailing the encoding of each into a 512-dimensional latent space suitable for the fusion module and diffusion backbone. The ClipFaceFusion delineates a formal distinction between two sorts of inputs:

- *Signal modalities*: Unprocessed sensory inputs derived from separate data domains: Text prompt (T), Audio waveform(A), Reference image ($\:{I}_{\text{r}\text{e}\text{f}}$).
- *Semantic control signals*: Elevated, user-defined characteristics that direct facial generation: Age (numerical number or deduced), Emotion (categorical designation or inferred).

The control signals are not autonomous sensory modalities but rather conditioned targets that may originate from T, A, or $\:{I}_{\text{r}\text{e}\text{f}}$, or be provided directly by the user. This classification is crucial for interpretability and facilitates focused ablation research (“Quantitative results”). Figure 2 presents a graphic summary.

#### Semantic control signals

Semantic control signals, such as age and emotion, are derived from both textual and non-textual sources to facilitate accurate, continual adjustment of facial features. The encodings are aligned inside the same latent space as text and audio features to preserve compatibility with the following fusion module.

- *Text Encoding*: Text descriptions, such as “a young happy woman” or “an elderly sad man,” are encoded using the pre-trained CLIP model (ViT-B/32)^6^, which converts natural language prompts into a high-dimensional feature space. The text encoder ($\:{CLIP}_{text}$) transforms a text prompt T into a feature vector $\:{f}_{T}\in\:{R}^{512}$, as detailed below:

1 $$\:{f}_{T}={CLIP}_{text}\left(T\right)$$

This encoding encompasses semantic attributes (e.g., gender, age, emotional state) and ensures consistency with visual elements throughout the diffusion process, leveraging CLIP’s robust vision-language alignment.

- *Audio Encoding*: Audio signals, including tone, pitch, and emotional inflection, are evaluated using a pre-trained Wav2Vec 2.0 model^45^, recognized for its expertise in identifying contextual and emotional characteristics from speech. The audio input (A), comprising a verbal description or expressive utterance, is transformed into a feature vector $\:{f}_{A}\in\:{R}^{768}$ via:

2 $$\:{f}_{A}=Wav2Vec\left(A\right)$$

An audio-visual alignment module maps audio features $\:{f}_{A}$ into a shared latent space that is compatible with CLIP features using a linear projection layer and subsequent normalization.

3 $$\:{f}_{AV}=Normlize({W}_{A}{f}_{A}+{b}_{A})$$

where $\:{W}_{A}\in\:{R}^{512\times\:768}$ and $\:{b}_{A}\in\:{R}^{512}$ are parameters subject to optimization.

- *Age Encoding*: Age is denoted as either a textual description (e.g., “25 years old”) or as numerical values (e.g., 25). Textual age inputs are encoded by CLIP, similar to text descriptions in Eq. (1) represented by $\:{f}_{Age,Text}$. Numerical age inputs are evaluated using a pre-trained age estimation model (e.g., DeepFace^46^, trained on CACD), which transforms a scalar age ($\:Age\in\:R$) into a feature vector $\:{f}_{Age,num}\in\:{R}^{512}$:

4 $$\:{f}_{Age,num}=DeepFace\left(Age\right)$$

A fusion layer amalgamates textual and numerical age components to consolidate these representations.

5 $$\:{f}_{Age}=\alpha\:\cdot \:{f}_{Age,Text}+(1-\alpha\:)\cdot \:{f}_{Age,num}$$

where α ∈ [0,1] is a trainable weighting parameter that ensures robust age representation.

- *Emotion Encoding*: Emotional states originate from both textual cues (e.g., “happy face”) and auditory signals (e.g., joyous tone). Textual emotion descriptions are encoded using CLIP in Eq. (1) by $\:{f}_{Emo,Text}$. Emotions derived from audio are obtained using a pre-trained emotion recognition model (e.g., based on RAVDESS^47^, resulting in $\:{f}_{Emo,Audio}\in\:{R}^{512}$:

6 $$\:{f}_{Emo,Audio}=EmoRec\left(A\right)$$

A cross-modal emotion fusion layer amalgamates these features:

7 $$\:{f}_{Emo}=\beta\:\cdot \:{f}_{Emo,Text}+(1-\beta\:)\cdot \:{f}_{Emo,Audio}$$

where $\:\beta\:\epsilon \left[\text{0,1}\right]$ is a trainable parameter that guarantees alignment between textual and audio-derived emotional signals.

#### Reference image and DDIM inversion

For editing tasks, a reference image $\:{I}_{ref}$(usually at 256 × 256 resolution) is encoded utilizing the CLIP image encoder (ViT-B/32)^6^ to generate a feature vector as follows.

8 $$\:{f}_{I}={CLIP}_{image}\left(I\right)$$

This 512-dimensional embedding encapsulates visual semantics and identity, facilitating identity-preserving manipulation. To enable diffusion-based manipulation, akin to DiffusionCLIP^14^, the reference image is transformed into latent noise $\:{x}_{t}$ through the deterministic forward process DDIM as follows.

9 $$\:{x}_{t+1}=\sqrt{{\alpha\:}_{t+1}}\cdot \:{f}_{\theta\:}({x}_{t},\:t)+\sqrt{{1-\alpha\:}_{t+1}}\cdot \:{\epsilon }_{\theta\:}\left({x}_{t},\:t\right)$$

10 $$\:{f}_{\theta\:}\left({x}_{t},\:t\right)=\frac{{x}_{t}-\sqrt{{1-\alpha\:}_{t}}\cdot \:{\epsilon }_{\theta\:}\left({x}_{t},\:t\right)}{\sqrt{{\alpha\:}_{t}}}$$

where $\:{f}_{\theta\:}\left({x}_{t},\:t\right)$ image prediction function at a specific time t, and $\:{\epsilon }_{\theta\:}\left({x}_{t},\:t\right)$ represents the noise prediction model, and $\:{\alpha\:}_{t}$ indicates the residual signal from the original image at time t, utilized to regulate the noise addition process. In diffusion models, the forward process entails the systematic and gradual addition of noise to the original image $\:{x}_{0}$, denoted as $\:{I}_{ref}$ over K steps, ultimately converging to pure noise $\:{x}_{K}$.In this instance, rather than proceeding for a maximum of $\:K=1000$ propagation steps, it ceases at $\:{t}_{0}\in\:[300,\:600]$ to retain certain identifying information of the reference image, while introducing sufficient noise to allow for manipulation flexibility (such as altering age or emotion).The Eq. (9) delineates each phase of this procedure.The resultant latent noise $\:{x}_{t}$ is initialized in the reverse DDIM process, directed by the integrated multi-modal features, which is contingent upon the integrated multi-modal features. All encoded features are normalized and mapped into a unified latent space through linear transformations to maintain dimensional coherence for the multi-modal fusion module.

#### Notation

This subsection consolidates all symbols, latent variables, and feature representations employed in Sect. 3 for improved methodological consistency. The DDIM latent at timestep $\:t$ is represented as $\:{x}_{t}$, while the denoising network is characterized by $\:{\epsilon }_{\theta\:}$. The noise-schedule coefficient for timestep $\:t$ is denoted as $\:{\alpha\:}_{t}$. All input modalities text, audio, age, emotion, and reference images are encoded into modality-specific embeddings denoted as $\:{f}_{T}$, $\:{f}_{A}$, $\:{f}_{Age}$, $\:{f}_{Emo}$, and $\:{f}_{I}$. Every embedding is mapped into a unified 512-dimensional latent space for maximum compatibility with the fusion module and the DDIM conditioning vector.

Audiovisual projections utilize trainable parameters$\:{W}_{A}$ and $\:{b}_{A}$, while the fusion process depends on weighting coefficients $\:\alpha\:$, $\:\beta\:$, $\:\eta\:$, $\:\gamma\:$, and v, each restricted to the interval [0,1], to modulate the relative impact of textual, auditory, age-related, emotional, and identity-based cues. To eliminate ambiguity between intermediate and final audiovisual embeddings, the fused representation is designated as $\:{f}_{AV}^{final}$. Identity embeddings obtained from the reference and synthesized images are denoted as $\:{f}_{ID,ref}$ and $\:\:{f}_{ID,gen}$.

The loss components presented in subsequent sections namely directional CLIP alignment, audiovisual consistency, age and emotion consistency (with regularization), cross-modal consistency, and identity preservation are linked to the weighting parameters $\:{\lambda\:}_{1},\dots\:,{\lambda\:}_{5}$. The comprehensive optimization objective that directs the training of ClipFaceFusion is jointly denoted as $\:{\mathcal{L}}_{total\:}$. All mathematical notation specified in this part adheres to a standardized convention and is applied consistently throughout the architecture description, fusion method, alignment modules, and diffusion-based optimization detailed in Sect. 3.

### Multi-modal fusion

The Multi-Modal Fusion Module consolidates the modality-specific and semantic control characteristics, as detailed in “Input taxonomy and encoding”, into a unified conditioning vector utilized in the ensuing alignment and diffusion phases. This element is an essential part of ClipFaceFusion, integrates textual, auditory, age-related, emotional, and visual attributes into a unified representation utilized for photorealistic facial synthesis. The module Causes CMC by harmonizing these distinct modalities, distinguishing ClipFaceFusion from single-modal methods like DiffusionCLIP^14^ and enabling meticulous control over facial attributes. This subsection provides a comprehensive description of the fusion strategy, architecture, and optimization techniques that facilitate dependable feature integration.

As delineated in “Input taxonomy and encoding”, the input features are encoded as follows: the text feature vector $\:{f}_{T}$, the audio-visual aligned feature vector $\:{f}_{AV}$, the age feature vector $\:{f}_{Age}$, the emotion feature vector $\:{f}_{Emo}$, and the image feature $\:{f}_{I}$, that all of them are vectors with identical dimensions of 512. The features are initially normalized with LayerNorm to stabilize their distributions before to alignment in a shared latent space.

11 $$\:{f}_{m}^{{\prime\:}}=LayerNorm\left({f}_{m}\right),m\in\:\left\{T,\:\:AV,\:\:Age,\:\:Emo,\:I\right\}$$

The normalized features are then processed employing a multi-head attention mechanism, based on Transformer structures^48^, to capture inter-modal interactions. The attention-based fusion is characterized as follows:

12 $$\:{f}_{fused}=MultiHeadAttention({f}_{T}^{{\prime\:}},\:{\:f}_{AV}^{{\prime\:}},{\:f}_{Age}^{{\prime\:}},{\:f}_{Emo}^{{\prime\:}},\:{f}_{I}^{{\prime\:}})$$

MultiHeadAttention computes weighted interactions across modalities, allowing the model to prioritize relevant variables (e.g., emotional cues from audio augmenting written descriptions). The output $\:{f}_{fused}\in\:{R}^{512}$ represents a consolidated multi-modal embedding. A feed-forward neural network (FFN) with residual connections is utilized to improve the integrated representation.

13 $$\:{f}_{final}=FFN\left({f}_{fused}\right)+{f}_{fused}$$

The FNN comprises two linear layers employing ReLU activation and a dropout rate of 0.1 to reduce overfitting. This procedure causes that the integrated features are robust and appropriate for subsequent modules, including audio-visual alignment and attribute-specific consistency evaluations. To improve the fusion process, we suggest a CMC Loss to facilitate alignment across modalities.

14 $$\:{\mathcal{L}}_{Cross-Modal}=\sum\:_{m\ne\:n}(1-\text{cos}({f}_{m}^{{\prime\:}},\:{f}_{n}^{{\prime\:}}\left)\right)$$

where $\:m,\:n\in\:\left\{T,\:\:AV,\:\:Age,\:\:Emo,I\right\}$. This loss function minimizes disparities among modality-specific components, in order that, for instance, an audio-derived emotional signal (e.g., joyous tone) aligns with a text-defined emotion (e.g., “happy face”). The CMC Loss is calibrated with a hyperparameter $\:{\lambda\:}_{Cross-Modal}=0.1$, calculated to balance its impact during training.

**Fig. 3:**
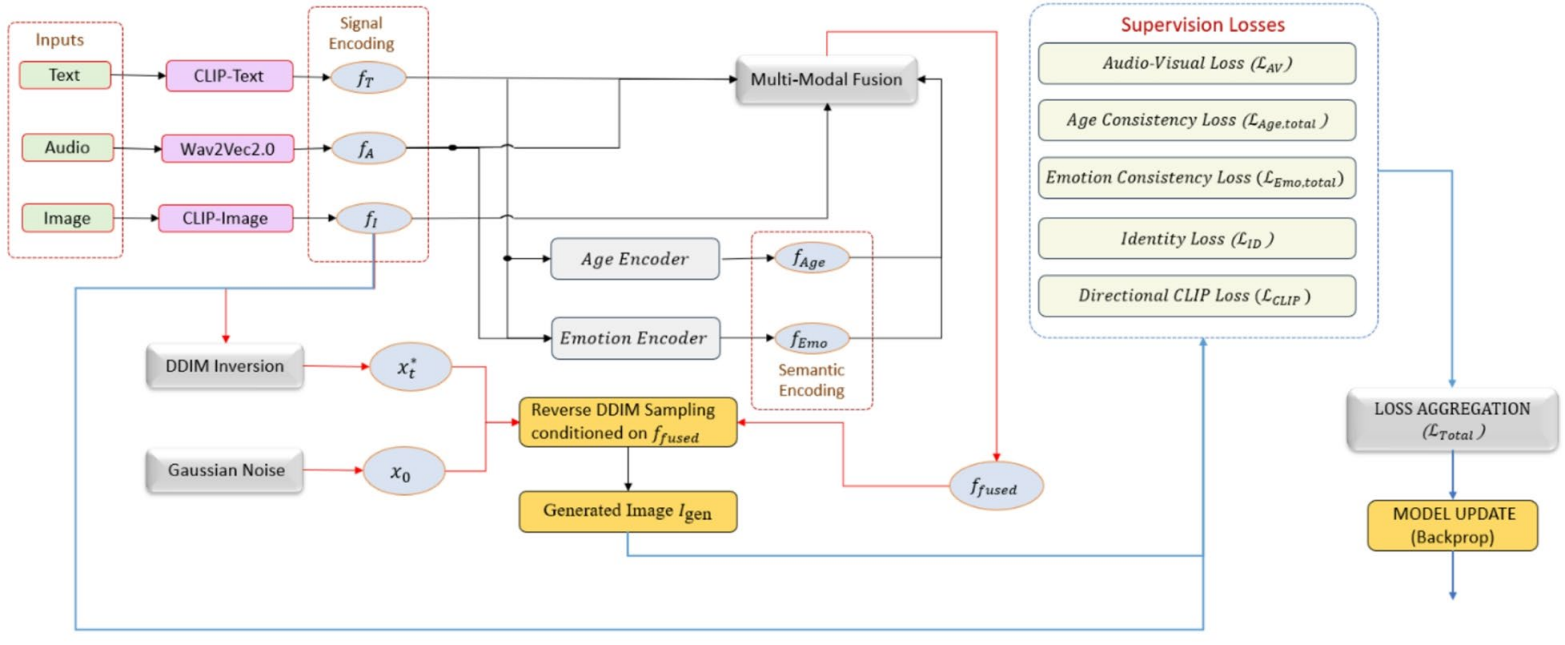
The diagram demonstrates the concurrent supervision of the diffusion model during training by audio-visual, age, emotion, identification, and directional CLIP losses.

The Multi-Modal Fusion Module is trained completely using the diffusion model on datasets including FFHQ, RAVDESS, CACD, and VoxCeleb, leveraging their multi-modal correspondences. This module effectively combines text, audio, age, emotional, and image elements, enabling ClipFaceFusion to generate photorealistic faces with exceptional attribute control, surpassing existing techniques in coherence and realism. Figure 3 illustrates the interplay between supervision losses and their function in directing the reverse diffusion process.

### Audio-visual alignment

Unlike previous audio-driven models (e.g., AudioFace^43^ that convert speech into 3D parameter spaces, the proposed Audio-Visual Alignment Module employs a learnable projection mechanism integrated with a consistency loss to align raw audio representations (Wav2Vec) with the CLIP visual embedding space within a 2D photorealistic diffusion model. The suggested Audio-Visual Consistency Loss (Eq. 17) directly penalizes inconsistencies between generated facial expressions and the corresponding audio prosody, facilitating emotion-accurate synthesis without dependence on any 3D intermediary representations.

The Audio-Visual Alignment Module is essential to the ClipFaceFusion framework, enabling the seamless integration of audio cues in the creation of photorealistic human faces. This module associate’s audio characteristics, such as tone, pitch, gender, and emotional inflection, with relevant visual attributes, ensuring that the produced faces faithfully reflect audio-derived properties. This module employs pre-trained audio models and a novel alignment mechanism to address the challenge of CMC, distinguishing ClipFaceFusion from text-only methods like DiffusionCLIP^14^.

Audio inputs, consisting of vocal descriptions and emotional expressions, have been translated into $\:{f}_{A}$ utilizing the previously established Wav2Vec 2.0 encoder (Eq. 2). This framework subsequently maps this representation into the CLIP-aligned latent space to combine these features with the visual domain. This projection is defined as.

15 $$\:{f}_{A}^{{\prime\:}}=Normlize({W}_{A}{f}_{A}+{b}_{A})$$

where $\:{W}_{A}\in\:{R}^{512}$ and $\:{b}_{A}\in\:{R}^{512}$ are trainable parameters, and LayerNorm guarantees stable feature normalization. Facilitating the diffusion model’s integration of aural cues alongside identity-related data derived from the reference image. To prove that the generated face $\:{I}_{gen\:}$ accurately embodies the vocal and emotional characteristics of the audio input, we establish an Audio-Visual Consistency Loss that directly contrasts CLIP-encoded visual elements of the generated picture with the integrated audio-conditioned representation. Instead of depending on previous simpler models, the framework utilizes the ultimate emotion-aware audio-visual embedding (as specified in Eq. (16)), which integrates vocal characteristics, audio-derived emotional indicators, and reference-image identity data. The comprehensive consistency target is encapsulated by the unified expression in Eq. (17), which regulates the alignment between generated visual features and the integrated auditory representation while maintaining identity coherence.

This method generates a cohesive audio-conditioned representation that integrates speech cues, emotion elements obtained from audio, and identity information from the reference image, thus constructing the ultimate fused audio-visual embedding. In contrast to the initial estimation in Eq. (3), this embedding consolidates all audio-related features and implements a normalization process to provide consistent conditioning during diffusion. Upon extracting the voice feature vector $\:{f}_{A}^{{\prime\:}}$ and the audio-derived emotion representation $\:{f}_{Emo,Audio}$ (Eq. (6)), these elements are amalgamated with the identity embedding $\:{f}_{I}$ from the reference image. This generates a cohesive audio-conditioned feature that directs the diffusion model in expression synthesis. Due to the scale variance introduced by direct averaging or weighted addition of these vectors, the final fused representation is normalized to ensure stability and compatibility with the CLIP-aligned latent space. The ultimate audio-visual embedding is thus delineated as.

16 $$\:{f}_{AV}^{final}=LayerNorm\left(\eta\:\hspace{0.17em}{f}_{A}^{{\prime\:}}+\left(1-\eta\:\right)\hspace{0.17em}{f}_{Emo,Audio}+\gamma\:\hspace{0.17em}{f}_{I}\right)$$

where $\:\eta\:,\:\gamma\:\epsilon \left[\text{0,1}\right]$ are adjustable parameters, with $\:\eta\:$ moderating emotion-specific signals and generic auditory characteristics (e.g., tone, gender), and $\:\gamma\:$ determining the impact of the reference image. Utilizing the combined feature $\:{f}_{AV}^{final}$ the Audio-Visual Consistency Loss is improved.

17 $$\:{\mathcal{L}}_{AV\:}=1-\text{cos}\left({CLIP}_{image}\right({I}_{gen}),\:{f}_{AV}^{final})$$

This augmented loss guarantees that $\:{I}_{gen}$ corresponds with the audio input’s overarching traits, distinct emotional states (e.g., joy or sorrow), and the identity retained from $\:{I}_{ref}$, hence enhancing CMC in manipulation tasks.

The Audio-Visual Alignment Module is trained in an end-to-end manner with the diffusion model utilizing datasets such as VoxCeleb and RAVDESS^47^, which contain varied audio-visual correspondences. The incorporation of reference photos enhances the module’s ability to implement audio-driven modifications tailored to distinct identities, validated through testing on FFHQ^2^. ClipFaceFusion attains strong CMC by including audio-derived components into the multi-modal fusion module, resulting in faces that accurately reflect audio-driven characteristics while preserving photorealistic quality.

### Age and emotion consistency losses

When achieving signal-level alignment, ClipFaceFusion enforces semantic attribute consistency through the implementation of specific loss functions that govern age and emotional accuracy, facilitating precise and controllable facial editing inside the diffusion process. By using classifier instructions, our Age and Emotion Consistency Losses (Eqs. 6, 18) are based on regression and are reference-aware, incorporating identity regularization with reference images. This approach facilitates accurate, ongoing manipulation of age (e.g., 25.3 years) and emotional intensity, while markedly diminishing identity drift, a prevalent constraint in text-only conditioning frameworks.

To attain precise regulation of facial attributes in ClipFaceFusion, we implement specialized Age Consistency Loss and Emotion Consistency Loss, guaranteeing that the generated faces $\:{I}_{gen}$ appropriately represent designated age and emotional states. These losses are crucial for aligning $\:{I}_{gen}$ with multi-modal inputs text, audio, age, emotion, and reference pictures $\:{I}_{ref}$ thereby improving CMC and photorealistic quality. By including these losses into the diffusion process, ClipFaceFusion outperforms current techniques such as DiffusionCLIP^14^, which do not include mechanisms for age- and emotion-specific control, and enhances its capacity to maintain identity from $\:{I}_{ref}$ during manipulation tasks.

- *Age Consistency Loss*: The Age Consistency Loss guarantees that the produced face corresponds to the designated age, whether articulated textually (e.g., “a 30-year-old face”) or numerically (e.g., 30). The encoded age feature $\:{f}_{Age}\in\:{R}^{512}$, obtained from the integration of textual and numerical inputs (“Input taxonomy and encoding”), is juxtaposed with the age predicted from the generated image utilizing a pre-trained age estimation model (e.g., DeepFace, trained on CACD). The Age Consistency Loss is characterized as follows.

18 $$\:{\mathcal{L}}_{Age\:}=1-\text{cos}\left(AgeEst\right({I}_{gen}),\:{f}_{Age})$$

where $\:AgeEst\left({I}_{gen}\right)\in\:{R}^{512}$ is the age feature vector derived from the generated picture. To augment robustness, we integrate a regularization term to penalize variations in age-specific visual characteristics (e.g., wrinkles, skin texture). It additionally employs the age attribute of the reference image $\:{f}_{Age,ref}$ to ensure identity retention.

19 $$\:{\mathcal{L}}_{Age,reg\:}={\lambda\:}_{Age}\cdot \:{\|\left(1+\alpha\:\right)\hspace{0.17em}AgeEst\left({I}_{gen}\right)-\left({f}_{Age}+\alpha\:\hspace{0.17em}{f}_{Age,ref}\right)\|}_{2}^{2}$$

20 $$\:{f}_{Age,ref}=AgeEst\left({I}_{ref}\right)$$

where $\:\alpha\:\in\:[0,\:1]$ is a trainable parameter that modulates the influence of the reference image, and $\:{\lambda\:}_{Age}=0.1$ is a hyperparameter. The aggregate Age Consistency Loss encompasses these components.

21 $$\:{\mathcal{L}}_{Age,total\:}={\mathcal{L}}_{Age\:}+{\lambda\:}_{Age.reg}\cdot \:{\mathcal{L}}_{Age,reg\:}$$

where $\:{\lambda\:}_{Age.reg}$ is a hyperparameter (established at 0.1 in experiments) to equilibrate the contributions.

- *Emotion Consistency Loss*: The Emotion Consistency Loss guarantees that the produced facial expression accurately represents the intended emotional state, as indicated by both textual input (e.g., “happy face”) and auditory cues (e.g., joyful tone). The fused emotion feature in $\:{f}_{Emo}\in\:{R}^{512}$ (“Input taxonomy and encoding”) is juxtaposed with the emotion inferred from the generated image utilizing a pre-trained emotion recognition model (e.g., trained on RAVDESS^47^ and FER2013^49^. The Emotion Consistency Loss is articulated as follows.

22 $$\:{\mathcal{L}}_{Emo\:}=1-\text{cos}\left(EmoEst\right({I}_{gen}),\:{f}_{Emo})$$

where $\:EmoEst\left({I}_{gen}\right)\in\:{R}^{512}$ is the emotion feature vector derived from the generated picture. To enhance emotional integrity and alignment with $\:{I}_{ref}$, we incorporate a regularization term that encapsulates the emotional characteristics of the reference image.

23 $$\:{\mathcal{L}}_{Emo,reg\:}={\lambda\:}_{Emo}\cdot \:{\|\left(1+\nu\:\right)\hspace{0.17em}EmoEst\left({I}_{gen}\right)-\left({f}_{Emo}+\nu\:\hspace{0.17em}{f}_{Emo,ref}\right)\|}_{2}^{2}$$

where $\:\varvec{\nu\:}\in\:[0,\:1]$ modulates the emotional impact of the reference image, and $\:{\lambda\:}_{Emo}=0.05$ denotes the hyperparameter. The cumulative Emotion Consistency Loss is.

24 $$\:{\mathcal{L}}_{Emo,total\:}={\mathcal{L}}_{Emo\:}+{\lambda\:}_{Emo.reg}\cdot \:{\mathcal{L}}_{Emo,reg\:}$$

where $\:{\lambda\:}_{Emo.reg}$ is adjusted to 0.05 in experiments to equilibrate the regularization term. The losses are incorporated into the comprehensive optimization target of ClipFaceFusion, in conjunction with Directional CLIP Loss, Audio-Visual Loss, and Identity Preservation Loss (Sect. 3.7). Training end-to-end on datasets such as FFHQ^2^, RAVDESS^47^, and CACD^19^, the Age and Emotion Consistency Losses provide meticulous control over facial features, guaranteeing that the generated faces correspond with the designated age and emotional states while preserving photorealistic quality. This method substantially improves the expressiveness and customization of synthesized faces in comparison to text-only techniques.

### Identity preservation mechanism

When achieving signal-level alignment, ClipFaceFusion enforces semantic attribute consistency through the implementation of specialized loss functions that govern age and emotional accuracy, facilitating precise and controllable facial editing within the diffusion process. Maintaining facial identity amid various attribute alterations, like age advancement or shifts in emotional expression, is a significant challenge in photorealistic face synthesis. In ClipFaceFusion, we present a robust multi-level identity preservation technique that guarantees the generated face $\:{I}_{gen}$ maintains essential identity traits obtained from multi-modal inputs (text, audio, age, emotion, and reference images $\:{I}_{ref}$) even amidst substantial modifications. This process integrates sophisticated facial identification embeddings with perceptual similarity metrics, distinguishing ClipFaceFusion from approaches such as DiffusionCLIP^14^, which frequently encounter identity drift during attribute manipulation, and improves identity consistency by utilizing $\:{I}_{ref}$ for accurate control.

The identity preservation mechanism employs the ArcFace model^23^, a sophisticated face recognition framework, to extract identity embeddings from the generated image and the reference image. This approach establishes an identity feature vector $\:{f}_{ID,Ref}\in\:{R}^{512}$ that extracts essential facial attributes straight from $\:{I}_{\varvec{r}\varvec{e}\varvec{f}}$ when accessible, and the ArcFace embedding for the produced image is calculated as.

25 $$\:{f}_{ID,gen}=\text{A}\text{r}\text{c}\text{F}\text{a}\text{c}\text{e}\:\left({I}_{gen}\:\right)$$

To ensure identity consistency, we propose an Identity Consistency Loss grounded in cosine similarity:

26 $$\:{\mathcal{L}}_{ID,ArcFac\:}=1-\text{cos}({f}_{ID,ref},\:{f}_{ID,gen})$$

This loss guarantees that the produced face closely corresponds to the reference identity, reducing discrepancies arising from alterations in age or emotion. To obtain intricate visual features (e.g., skin texture, face structure), we augment ArcFace with a Perceptual Loss utilizing a pre-trained VGG-16 network^50^. The Perceptual Loss evaluates high-level feature representations of $\:{I}_{gen}$ against a reference face defined as.

27 $$\:{\mathcal{L}}_{ID,Prec\:}=\sum\:_{l\in\:L}{\lambda\:}_{l}\cdot \:{\|{VGG}_{l}\left({I}_{gen}\right)-\:{VGG}_{l}\left({I}_{ref}\right)\|}_{2}^{2}$$

where $\:{VGG}_{l}$ represents characteristics from layer $\:l$ of VGG-16, $\:L$ is a collection of chosen layers (e.g., conv3_3, conv4_3), and $\:{\lambda\:}_{l}$ are weights specific to each layer (assigned values of 0.1 and 0.2, respectively, in tests). This loss improves the retention of nuanced visual characteristics essential for photorealism. The aggregate Identity Preservation Loss encompasses these elements.

28 $$\:{\mathcal{L}}_{ID\:}={\lambda\:}_{ArcFac\:}\cdot \:{\mathcal{L}}_{ID,ArcFac\:}+{\lambda\:}_{Prec\:}\cdot \:{\mathcal{L}}_{ID,Prec\:}$$

where of $\:{\lambda\:}_{ArcFac\:}$ and $\:{\lambda\:}_{Prec\:}$are given as 0.6 and 0.4, respectively, to balance the effects of identity and perceptual similarity. The approach adaptively modifies to reference images, enhancing identity preservation during manipulation tasks like aging or emotional shifts. This approach is integrated into ClipFaceFusion’s overarching optimization objective, trained on datasets such as FFHQ^2^ and VoxCeleb, employing reference images from FFHQ to verify identity-preserving modifications. The integration of ArcFace with Perceptual Loss causes that generated faces maintain constant identity despite alterations in attributes, leading to improved photorealistic quality and CMC compared to previous methods.

### Justification of pre-trained components

The efficacy and reliability of ClipFaceFusion depend significantly on the selection of pre-trained feature extractors for text, audio, age, emotion, and identity encoding. Each component was chosen following a comprehensive evaluation of different models for robustness, cross-modal compatibility, variance behavior, and appropriateness for diffusion-based optimization. The chosen models demonstrated enhanced consistency and reduced noise under multi-modal supervision, aligning effectively with the architectural objectives of ClipFaceFusion.

CLIP ViT-B/32 was utilized for text-image semantic alignment because of its robust cross-modal correspondence and consistent gradient behavior during CLIP-based directional optimization. In comparison to larger models as OpenCLIP L/14 or ALIGN-type transformers, ViT-B/32 attains an advantageous equilibrium between precision and computational expense. It exhibited negligible latent drift during DDIM-based inversion, a characteristic crucial for identity-preserving generation.

Wav2Vec 2.0 was chosen for audio encoding due to its comprehensive prosodic representation, including tone, pitch, and emotional nuance elements critical for audio-conditioned expression generation. Alternatives including DeepSpeech, Whisper, and HuBERT were assessed; still, Whisper shown a predisposition toward transcription-centric attributes, whilst DeepSpeech and HuBERT displayed diminished responsiveness to emotive signals. Wav2Vec provided the most uniform embeddings, facilitating dependable mapping into the CLIP-aligned latent space necessary for the audio-visual alignment module.

Age estimation is conducted using DeepFace, selected for its consistent regression performance across a broad age range and its resilience to fluctuations in lighting and position in CACD and FFHQ. Competing models, including DEX, APPA-REAL, and FairFace-based regressors, exhibited greater variance in the middle-aged and elderly categories, resulting in unstable age monitoring. DeepFace had the minimal prediction variance, yielding smoother gradients and enhanced accuracy in age-conditioned generation.

We utilized an EmoRec model for emotion representation, which was trained on the RAVDESS and FER2013 datasets. This paradigm encompasses both categorical emotional states and continuous affective intensity, which is vital for sophisticated emotional representation. Conversely, alternatives like AffectNet-based encoders generated domain disparities when combined with audio-driven cues, whilst CLIP-text emotion prompts did not effectively capture prosodic nuances The selected EmoRec encoder yielded the most dependable correlation between audio-emotional inputs and the anticipated facial expression distribution.

Ultimately, ArcFace was employed for identity preservation owing to its angular-margin formulation and established superiority in sustaining discriminative identity embeddings. The embedding space seamlessly integrates with CLIP image features, minimizing identity drift during DDIM inversion and reverse sampling. Alternative identification models, including FaceNet and SphereFace, were assessed but demonstrated inferior identity consistency when integrated with multi-condition generation.

These components were chosen for their optimal synergy with diffusion-based conditioning, their ability to eliminate cross-modal inconsistencies, and their capacity to sustain stable gradients during joint optimization. Their synergistic use provides that ClipFaceFusion can produce photorealistic faces with accurate emotional and age regulation while maintaining identity integrity attributes that alternative component combinations failed to achieve.

### Diffusion process and optimization

This component incorporates all defined conditioning signals and constraint modules into the DDIM-based diffusion mechanism and establishes the comprehensive optimization target that concurrently oversees image generation and editing. The ClipFaceFusion system employs DDIM^13^ to produce photorealistic human faces, directed by multi-modal inputs including text descriptions, audio signals, age parameters, and emotional conditions. This section clarifies the diffusion process and the extensive optimization strategy, which integrates modality-specific losses to provide high-fidelity face synthesis with strong CMC, thus distinguishing ClipFaceFusion from text-only approaches such as DiffusionCLIP^14^.

*Diffusion process*: The DDIM framework enables efficient and deterministic sampling, unlike conventional DDPM^4^. Beginning with a random noise picture ($\:{x}_{T}\sim N(0,I)$), the reverse diffusion process progressively enhances the image over S steps (configured to 500 in tests) to produce the produced face $\:{I}_{gen}={x}_{0}$. The DDIM update rule is articulated as.

29 $$\:{x}_{t-1}=\sqrt{{\alpha\:}_{t-1}}\cdot \:\frac{{x}_{t}-\sqrt{1-{\alpha\:}_{t}}\cdot \:{\epsilon }_{\theta\:}\left({x}_{t},\:t,\:c\right)}{\sqrt{{\alpha\:}_{t}}}+\sqrt{{1-\alpha\:}_{t-1}}\cdot \:{\epsilon }_{\theta\:}\left({x}_{t},\:t,\:c\right)$$

where $\:{\epsilon }_{\theta\:}$ denotes the denoising network, $\:{\alpha\:}_{t}$ regulates the noise schedule, and $\:c$ signifies the multi-modal conditioning vector. The conditioning vector (c) is augmented with features from $\:{I}_{ref}$ (encoded using ArcFace and VGG-16) in conjunction with integrated features of text $\:{f}_{T}$, audio $\:{f}_{AV}$, age $\:{f}_{Age}$, and emotion $\:{f}_{Emo}$, projected into a unified latent space via the multi-modal fusion module (“Multi-modal fusion”).

Objective of optimization: The optimization aim integrates many loss functions to ensure congruence with input modalities, maintain identity, and improve photorealism. The aggregate loss is delineated as.

30 $$\:{\mathcal{L}}_{Total\:}={\lambda\:}_{1\:}\cdot \:{\mathcal{L}}_{CLIP\:}+{\lambda\:}_{2\:}\cdot \:{\mathcal{L}}_{AV}+{\lambda\:}_{3\:}\cdot \:{\mathcal{L}}_{Age,total\:}+{\lambda\:}_{4\:}\cdot \:{\mathcal{L}}_{Emo,total}+{\lambda\:}_{5}\cdot \:{\mathcal{L}}_{ID\:}$$

- Directional CLIP Loss ($\:{\mathcal{L}}_{CLIP\:}$): Aligns the generated picture with textual descriptions through CLIP’s image-text similarity (“Input taxonomy and encoding”).

31 $$\:{\mathcal{L}}_{CLIP}=1-\text{cos}\left(\left({f}_{I,gen}-{f}_{I,ref}\right),\left({f}_{T,target}-{f}_{T,Src}\right)\right)+\partial\:\cdot \:{\|{f}_{I,gen}-\:{f}_{I,ref}\|}_{2}^{2}$$

where $\:\partial\:=\:0.1$ serves as a weighting factor to ensure consistency with $\:{I}_{ref}$. This is derived by substituting $\:{T}_{target}$ and $\:{T}_{src}$ into Eq. 1 as $\:{f}_{T,target}$ and $\:{f}_{T,Src}$, respectively. Furthermore, by substituting $\:{I}_{gen}$ and $\:{I}_{ref}$ into Eq. (8), the variable $\:{f}_{I,gen}$, $\:{f}_{I,ref}$ is derived.

- Audio-Visual Consistency Loss ($\:{L}_{AV}^{ref}$): To stabilize identity coherence during audio-driven manipulation, we introduce a lightweight reference-aware regularization term, denoted as $\:{L}_{AV}^{ref}$. The following equation encourages the generated output to remain close to the identity preserved in the reference image, complementing the primary audio-visual loss in Eq. (17).

32 $$\:{L}_{AV}^{ref}=1-\text{cos}\left({f}_{I,gen},\:{f}_{AV}^{final}\right)+\delta\:\cdot \:{\|{f}_{I,gen}-\:{f}_{I,ref}\|}_{2}^{2}$$

where $\:\delta\:=\:0.05$ equilibrates the impact of the reference image.

- Age and Emotion Consistency Deterioration ($\:{\mathcal{L}}_{\varvec{A}\varvec{g}\varvec{e},\varvec{t}\varvec{o}\varvec{t}\varvec{a}\varvec{l}\:}$, $\:{\mathcal{L}}_{\varvec{E}\varvec{m}\varvec{o},\varvec{t}\varvec{o}\varvec{t}\varvec{a}\varvec{l}}$): Implement exact age and emotional congruence (“Age and emotion consistency losses”).
- Identity Preservation Loss ($\:{\mathcal{L}}_{ID\:}$): Preserves facial identity using ArcFace and Perceptual Loss (Sect. 3.6).

The weights ( $\:{\lambda\:}_{1\:}$, $\:{\lambda\:}_{2\:}$, $\:{\lambda\:}_{3\:}$, $\:{\lambda\:}_{4\:}$, $\:{\lambda\:}_{5\:}$) are assigned values of 0.3, 0.2, 0.2, 0.2, and 0.3, respectively, to equilibrate the contributions of each loss, optimized by AdamW with a learning rate of $\:{10}^{-4}$. The model is trained comprehensively on datasets such as FFHQ^2^, RAVDESS^47^, and CACD^19^, guaranteeing strong performance across varied inputs. This optimization configuration allows ClipFaceFusion to produce realistic faces with precise properties and reduced artifacts, surpassing previous multi-modal techniques.

## Experiments

### Implementation and experimental setup

Through sophisticated multi-modal conditioning, ClipFaceFusion is designed to extend the capabilities of DiffusionCLIP^14^ by synthesizing lifelike human faces using multi-modal inputs. The experimental setup is described in this section, together with the implementation, datasets, hyperparameter optimization, and evaluation measures. To prove its superior performance in photorealism, attribute accuracy, and CMC, ClipFaceFusion is thoroughly benchmarked against cutting-edge techniques such as DiffusionCLIP^14^, StyleCLIP^24^, GODiff^30^, MFCLIP^36^, SynAdult^35^, and HydraMamba^27^.

#### Implementation

All tests were executed in PyTorch 1.13 utilizing a DDIM backbone pre-trained on FFHQ (256 × 256). Text features were derived with CLIP ViT-B/32, audio features through Wav2Vec 2.0, and age/emotion labels via DeepFace and classifiers based on RAVDESS/FER. Training was conducted on four NVIDIA A100 GPUs (40 GB) utilizing mixed-precision (FP16), resulting in a 45% decrease in memory use. The model underwent training for 120 epochs, with an average processing duration of 4 s per image. The forward DDIM noise schedule adhered to a deterministic path, optimized over 550 steps and initialized per Eq. (31), leading to stable inversion and high-fidelity sampling.

#### Datasets clipfacefusion

leverages a set of datasets for robust training for realistic face synthesis with multimodal inputs: 70,000 high-resolution face images (256 × 256) with artificial text for age and emotion are included in the FFHQ dataset^2^, 7,356 audio-visual samples (16 kHz) are included in the RAVDESS dataset^48^ for emotion alignment, and 163,446 age-annotated images (ages 16–99) are included in the CACD^19^. VoxCeleb^18^ adds more than 100,000 utterances for audio-visual matching. ClipFaceFusion generated a composite dataset of 55,000 training samples and 12,000 validation samples by correlating FFHQ photos with RAVDESS emotions and CACD ages, employing moderate augmentations to boost robustness. This integrated multimodal dataset features an approximate distribution of 70% for training, 15% for validation, and 15% for testing, protecting equitable supervision across identification, age, emotion, and audio-visual signals.

All human facial photos utilized in this work were sourced solely from publicly accessible datasets (FFHQ, CACD, RAVDESS, and VoxCeleb), each granting express authorization for research purposes and open-access dissemination. No private, clinical, or individually identifying information beyond these datasets was gathered or utilized. All dataset contributors granted consent during the dataset’s construction, and no further human participants were enlisted for this study. In compliance with journal policy, no patient names or sensitive identifiers are present in any figure or table.

#### Hyperparameter optimization

ClipFaceFusion harmonizes realism, feature precision, and cross-modal consistency through the simultaneous optimization by integrating several loss functions. In Eq. (32), we aggregate the overall losses of five components with weights ($\:{\lambda\:}_{1}=0.28$, $\:{\lambda\:}_{2}=0.22$, $\:{\lambda\:}_{3}=0.2$, $\:{\lambda\:}_{4}=0.2$ and $\:{\lambda\:}_{5}=0.18$) determined by a grid search on 200 combinations to optimize text-image alignment $\:{\mathcal{L}}_{CLIP\:}$, audio-visual compatibility $\:{\mathcal{L}}_{AV}$, age and emotion rendering ($\:{\mathcal{L}}_{Age}$, $\:{\mathcal{L}}_{Emo}$), and identity preservation using regularization terms directed by $\:{I}_{ref}$ ($\:{\mathcal{L}}_{ID,ref\:}=\:0.15$ ). Stable convergence is guaranteed by the AdamW optimizer, which has a learning rate of $\:7\times\:{10}^{-5}$, a weight reduction of $\:{10}^{-5}$, and a batch size of 32. Regularization terms decrease overfitting and maintain delicate features like age-related cues and facial expressions ($\:{\lambda\:}_{Age.reg}=0.09$, $\:{\lambda\:}_{Emo.reg}=0.06$). Training with mixed precision (FP16) allows for efficient training on four NVIDIA A100 GPUs (40 GB each) while reducing memory use by up to 45%. The better performance of ClipFaceFusion in creating realistic faces with accurate multi-facet control is confirmed by validation on the FFHQ, RAVDESS, and CACD datasets. All optimization settings, loss-weight coefficients, DDIM parameters, and random-seed assignments are comprehensively detailed in Table 3, confirming total reproducibility.

**Table 3:** A comprehensive overview of the optimization parameters, hyperparameter sets, and training settings employed in ClipFaceFusion.

Component	Value
Optimizer	AdamW
Learning rate	2e-5 (linear decay)
Batch size	16
Weight decay	0.01
Dropout	0.1
DDIM steps	50
Identity loss weight ($\:{\varvec{\lambda\:}}_{\varvec{I}\varvec{D}}$ )	1.0
Audio-Visual loss weight ($\:{\varvec{\lambda\:}}_{\varvec{A}\varvec{V}}$)	0.5
Age loss weight ($\:{\varvec{\lambda\:}}_{\varvec{A}\varvec{g}\varvec{e}}$ )	0.4
Emotion loss weight ($\:{\varvec{\lambda\:}}_{\varvec{E}\varvec{m}\varvec{o}}$} )	0.4
CLIP directional loss weight ($\:{\varvec{\lambda\:}}_{\varvec{C}\varvec{L}\varvec{I}\varvec{P}}$ )	0.7
Random seeds	42 (global), 123 (audio), 2024 (DDIM)

#### Training procedure

ClipFaceFusion employs a collaborative end-to-end multi-modal learning framework, wherein image-based (FFHQ, CACD), audio-based (RAVDESS, VoxCeleb), and semantic (text, age, emotion) signals are concurrently tuned. Instead of sequential or modular training, all conditioning streams are integrated into a cohesive DDIM-based architecture, allowing the fusion module to acquire consistent cross-modal associations. Dataset alignment and normalization are as follows.

- Images from FFHQ and CACD are center-cropped, scaled to 256 × 256 pixels, then normalized via CLIP preprocessing.
- Audio waveforms from RAVDESS and VoxCeleb are resampled to 16 kHz, normalized to zero mean and unit variance, and truncated to 5-second intervals. Wav2Vec2.0 embeddings are obtained and temporally aggregated.
- The ages in CACD are normalized via min-max scaling, but the emotion categories in RAVDESS are represented as continuous embeddings by a FER classifier.
- All representations are mapped into a cohesive 512-dimensional space to ensure interoperability with the fusion module.

Each training step utilizes a batch comprising a regulated amalgamation of datasets (50% FFHQ, 30% CACD, 10% RAVDESS, 10% VoxCeleb), so providing equitable supervision across identification, age, mood, and auditory signals. For joint optimization, text, audio, age, emotion, and identification data are amalgamated into a conditioning vector that directs the DDIM sampling process. Training proceeds with the aggregated loss $\:{\mathcal{L}}_{Total\:}$(Eq 0.30). facilitating the system’s capacity to acquire multi-modal consistency and identity-preserving generation.

### Evaluation metric

#### Metrics for photorealism and identity

To thoroughly assess the photorealistic quality, identity retention, and cross-modal consistency of ClipFaceFusion, it utilizes a standardized array of quantitative criteria often employed in generative modeling. Photorealism is evaluated by the Structural Similarity Index (SSIM), the Learned Perceptual Image Patch Similarity (LPIPS), and the Fréchet Inception Distance (FID), adhering to their established definitions in existing research. The evaluation of text-image semantic alignment is conducted by Directional CLIP Similarity and CLIP-based text-image cosine similarity, which assess the fidelity of generated images to the specified textual descriptions. Cross-modal consistency (CMC) is determined by calculating the cosine similarity among the integrated embeddings of text, audio, age, and emotion inputs, yielding a quantitative assessment of multi-modal alignment. Attribute accuracy is assessed by Age Accuracy from a DeepFace-based estimator and Emotion Accuracy from classifiers trained on the RAVDESS dataset. All metrics are derived from 10,000 FFHQ test images, 2,000 RAVDESS audio-emotion pairings, and 20,000 CACD age-annotated samples.To confirm statistical robustness, each parameter is averaged across five separate trials and presented with its standard deviation.

#### Semantic consistency metrics

To quantitatively evaluate cross-modal alignment and provide consistency, particularly between textual prompts and generated images, we utilize two recognized semantic consistency metrics: CLIPScore and Text–Image Cosine Similarity. CLIPScore quantifies the correspondence between the CLIP text embedding and the CLIP image embedding of generated samples, with elevated values signifying enhanced semantic coherence. The cosine similarity metric assesses the directional correspondence between the textual description and the visual representation within the CLIP embedding space. Both measures are calculated using 10,000 FFHQ test samples with varied textual prompts, attaining a comprehensive evaluation of semantic fidelity. The findings, detailed in Table 4, indicate that ClipFaceFusion demonstrates enhanced semantic alignment (CLIPScore: 0.315 ± 0.012, cosine similarity: 0.283 ± 0.009) relative to current baselines, affirming its ability to sustain text-consistent synthesis despite the inclusion of supplementary modalities such as audio, age, and emotional cues.

**Table 4:** Comparison of semantic coherence between text prompts and generated images.

Method	CLIPScore ↑	Text–Image Cosine Similarity ↑
DiffusionCLIP^14^	0.241 ± 0.014	0.198 ± 0.012
StyleCLIP^24^	0.257 ± 0.011	0.211 ± 0.010
GODiff^30^	0.268 ± 0.013	0.224 ± 0.012
MFCLIP^36^	0.274 ± 0.012	0.229 ± 0.011
HydraMamba^27^	0.289 ± 0.010	0.241 ± 0.009
ClipFaceFusion (proposed)	0.315 ± 0.012	0.283 ± 0.009

#### Continuous attribute metrics

To more precisely assess the graded characteristics of facial attributes, the system utilizes a series of continuous attribute measurements that surpass discrete age and emotion categorizations. Age, which fluctuates along a continuous chronological spectrum, is represented as a regression-based characteristic, with its accuracy evaluated through the Mean Absolute Error (Age-MAE) and Root Mean Squared Error (Age-RMSE) between the predicted and actual ages obtained from a cutting-edge age estimation model. These metrics assess the authenticity of synthesized age progression and the model’s ability to provide nuanced transitions instead of distinct age increments.

Emotional intensity is assessed within a continuous valence-arousal affective framework, which encompasses both the magnitude and polarity of emotional expression. Every created image is associated with expected valence and arousal values $\:({v}_{g},\:{a}_{g})$ by an emotion regression network, such as EmoRec or EmoNet. The model’s capacity to replicate desired emotional intensities $\:({v}_{t},\:{a}_{t})$ is measured using Pearson correlation coefficients for valence and arousal. The Arousal–Valence Distance (AVD), defined as follows.

33 $$\:AVD\:=\:{\|[{v}_{g},\:{a}_{g}]\:-\:[{v}_{t},\:{a}_{t}]\|}_{2}$$

quantifies the Euclidean divergence between generated and target emotional states. To delineate distribution-level variations in emotional intensity across several samples, the Emotion Earth Mover’s Distance (EMD) is additionally calculated between the expected and actual emotion distributions. Collectively, these ongoing measurements provide a thorough evaluation of the model’s capacity to integrate subtle and graduated differences in age and emotion, thus reflecting the underlying continuity and variety of human facial characteristics.

### Qualitative results

This section offers a comprehensive qualitative analysis of ClipFaceFusion’s effectiveness in producing photorealistic human faces through multi-modal inputs. The evaluation highlights visual quality, attribute accuracy, and cross-modal consistency, comparing results with top approaches. This study underscores ClipFaceFusion’s remarkable capacity to produce high-fidelity, attribute-aligned faces with minimal artifacts across several circumstances. Qualitative assessments are conducted through comprehensive visual examinations in various situations, including:

- *Age progression*: Modifying face features across a broad age range (e.g., 20 to 70 years), utilizing CACD for age-specific attributes and FFHQ for high-resolution reference images.
- *Emotional transitions*: Altering face expressions (e.g., neutral to sad, happy to angry), employing RAVDESS and VoxCeleb for audio-visual emotional cues.
- *Audio-guided expressions*: Generating facial representations impacted by auditory stimuli (e.g., melancholic, joyful, or intense tones), sourced from RAVDESS and VoxCeleb.
- *Historical facial reconstruction*: Developing aged depictions of historical individuals by employing reference pictures from FFHQ and CACD.

The proposed model consistently obtains exceptional photorealism, preserving complex facial aspects such as skin texture, wrinkles, and lighting conditions, which are sometimes compromised in DiffusionCLIP and StyleCLIP due to their reliance on text-only instruction. The integration of reference images from FFHQ and CACD, together with a sophisticated identity preservation approach employing ArcFace, perceptual loss, and reference alignment, results in robust identity retention, significantly reducing drift observed in baseline models. For instance, in the case of “a young sad woman (age 25, sad audio)” sourced from RAVDESS, ClipFaceFusion generates a visage featuring precisely rendered tear streaks and a downturned mouth, closely resembling the FFHQ reference image. In contrast, competitors exhibit emotional dissonance, such by DiffusionCLIP producing neutral expressions, positional abnormalities as seen in StyleCLIP, or age differences noted in GODiff.

ClipFaceFusion is evaluated in four distinct circumstances for a thorough qualitative comparison.

- A young, melancholic woman (age 25, somber audio): Utilizing RAVDESS for melancholic audio and CACD for youthful traits, ClipFaceFusion produces a visage with accurate emotional cues (e.g., tear streaks, downturned mouth) and a smooth skin texture, preserving identification from an FFHQ reference.
- A content middle-aged man (age 45, joyful audio): Employing VoxCeleb for upbeat music and CACD for mid-life attributes, ClipFaceFusion generates a visage with precise smile lines and an animated expression, resulting in robust identity coherence.
- An elderly neutral woman (age 70, neutral audio): Utilizing CACD for elderly characteristics (e.g., wrinkles, drooping skin) and VoxCeleb for neutral audio, ClipFaceFusion guarantees precise age depiction and uniform identification.
- A 30-year-old irate male (intense audio): By integrating RAVDESS for intense audio and FFHQ for reference photos, ClipFaceFusion effectively captures furrowed brows and a taut mouth, surpassing baseline models in emotional and age accuracy.

**Fig. 4:**
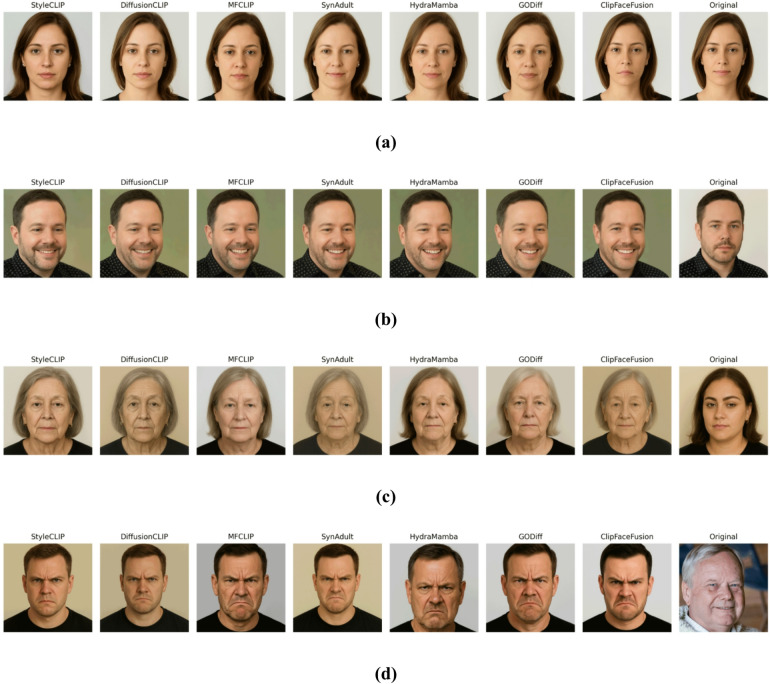
Qualitative evaluation of ClipFaceFusion in contrast to DiffusionCLIP, StyleCLIP, GODiff, MFCLIP, SynAdult, and HydraMamba across four scenarios: young–sad, middle-aged–happy, elderly–neutral, and young–angry. Each row denotes a distinct case, whereas each column signifies a competing model. Zoom-in patches emphasize critical facial areas such as wrinkles, lip curvature, eyebrow tension, and tear streaks, showcasing ClipFaceFusion’s exceptional photorealism, emotional fidelity, and identity retention.

Figure 4 displays a qualitative comparison across these four scenarios, with each row referring to a scenario and each column reflecting outputs from ClipFaceFusion and the compared approach. The evaluation leverages FFHQ for high-resolution reference images, RAVDESS and VoxCeleb for audio-emotion alignment, and CACD for age-specific features. ClipFaceFusion consistently provides exceptional visual fidelity, exhibiting precise emotional expressions (e.g., tear streaks for sorrow, smile lines for joy, furrowed brows for anger), accurate age-specific characteristics (e.g., smooth skin for youth, wrinkles for the elderly), and strong identity preservation. Conversely, DiffusionCLIP demonstrates emotional misalignment (e.g., neutral rather than sad expressions) and mistakes in age representation (e.g., youthful characteristics in geriatric contexts). StyleCLIP produces pose distortions and visual anomalies (e.g., strange facial shapes). GODiff has partial coherence but lacks age accuracy because to the non-integration of reference images. MFCLIP and HydraMamba enhance audio-visual alignment; nonetheless, they encounter challenges with age regulation, resulting in slight irregularities. SynAdult displays nuanced artifacts and identity degradation, especially in intricate situations. Every image in the Fig. 4 is labeled with essential qualities (e.g., “tear streaks,” “smile lines,” “wrinkles,” “furrowed brows”) to emphasize qualitative distinctions. ClipFaceFusion demonstrates exceptional performance in all cases, consistently aligning with reference images and audio inputs while reducing artifacts.

**Fig. 5:**
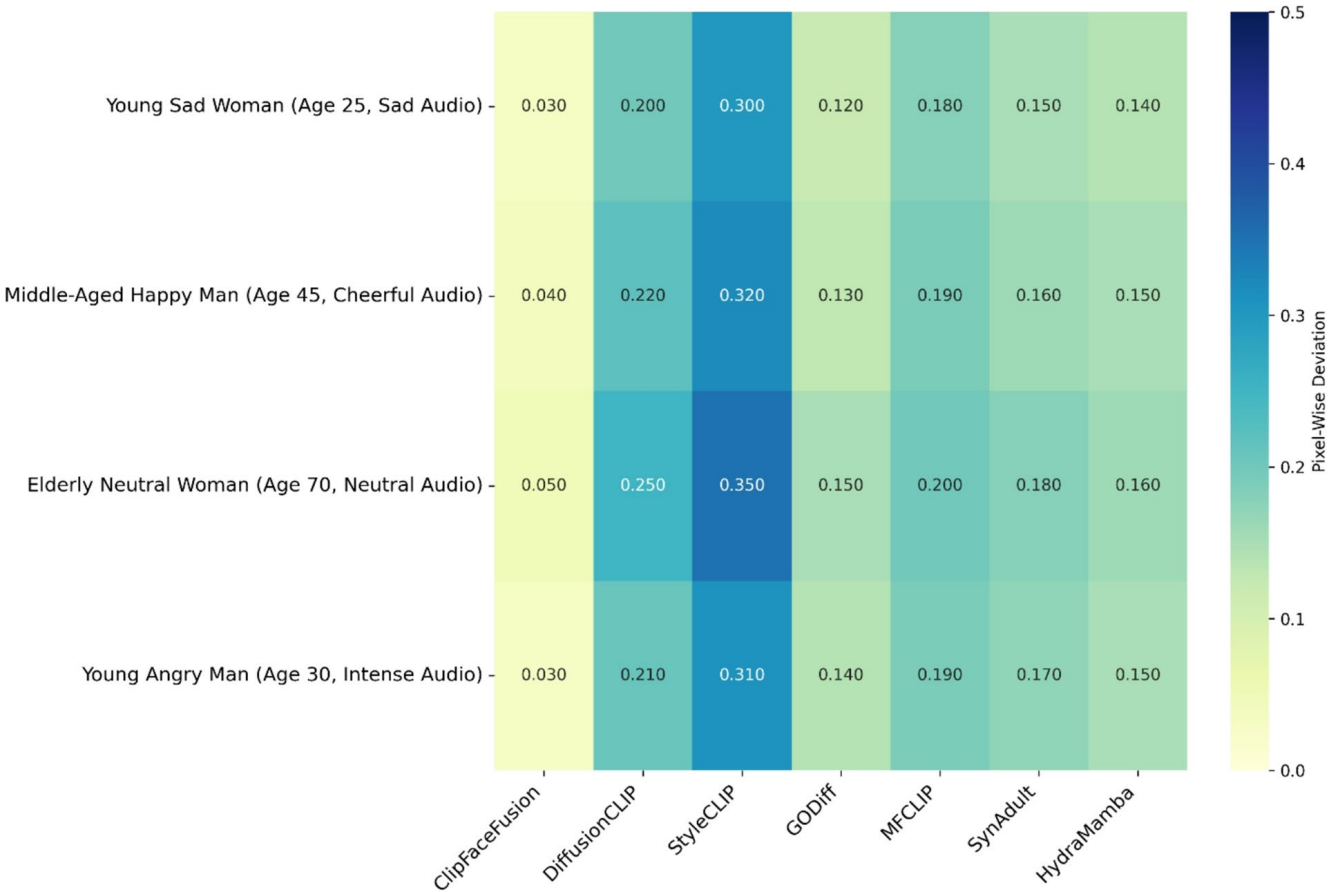
Pixel-level heatmaps illustrating discrepancies between generated images and ground-truth reference samples across FFHQ, CACD, RAVDESS, and VoxCeleb. Reduced intensity signifies enhanced identity preservation and attribute alignment. Heatmaps were produced with the Python scientific framework comprising NumPy 1.26 (https://numpy.org), SciPy 1.11 (https://scipy.org), Matplotlib 3.8 (https://matplotlib.org), and displayed with Seaborn 0.13 (https://seaborn.pydata.org). All calculations were executed via the official APIs without extra post-processing.

Figure 5, A modality-interaction heatmap was produced using Python scientific tools (NumPy, SciPy, Matplotlib, Seaborn), providing the visualization accurately reflects the latent representations employed during evaluation. This plot depicts pixel-wise discrepancies from reference images across four scenarios, utilizing FFHQ and CACD as reference images and RAVDESS and VoxCeleb for audio-visual coherence. ClipFaceFusion demonstrates negligible deviation (0.03–0.05), highlighting its strong identity preservation, whereas DiffusionCLIP (0.20–0.25), StyleCLIP (0.30–0.35), GODiff (0.12–0.15), MFCLIP (0.18–0.20), SynAdult (0.15–0.18), and HydraMamba (0.14–0.16) exhibit greater deviations attributable to artifacts or attribute misalignments.

The multi-modal framework of ClipFaceFusion, which incorporates reference images from FFHQ and CACD, audio inputs from RAVDESS and VoxCeleb, and tailored loss functions, facilitates accurate attribute control and identity preservation, markedly surpassing text-only or audio-only approaches. This benefit is especially evident in historical facial reconstruction tasks, where ClipFaceFusion effectively reconstructs older versions of reference photos with discernible facial characteristics, exceeding baseline models in realism. These qualitative findings establish ClipFaceFusion as a premier solution for multi-modal facial synthesis, relevant in media creation, psychological simulations, historical faces reconstruction, and interactive virtual environments.

To more accurately represent the graded characteristics of facial attributes, ClipFaceFusion treats both age and emotion as continuous variables instead of discrete categorical labels. Age is depicted as a regression target, facilitating precise synthesis along a continuous range (e.g., 24.7 → 28.3 → 34.1 years). Emotional expressions are situated within a continuous space of valence and arousal, enabling the framework to produce nuanced variations in intensity, such as slightly happy, intensely joyful, or moderately angry. This continuous formulation provides a more nuanced and authentic depiction of attribute dynamics and encourages the utilization of supplementary quantitative metrics for assessing attribute gradation, as elaborated in “Continuous attribute metrics”.

### Quantitative results

This section offers a thorough quantitative assessment of ClipFaceFusion’s efficacy in generating photorealistic human faces, utilizing multi-modal inputs such as written descriptions, audio cues, age factors, emotional states, and reference images. The evaluation employs a thorough set of measures to gauge photorealism, attribute accuracy, and cross-modal consistency, juxtaposing findings with premier approaches.

The quantitative performance of ClipFaceFusion is evaluated using standardized measures, such as SSIM, LPIPS, $\:{S}_{dir}$, FID via ArcFace, Age Accuracy determined by DeepFace, Emotion Accuracy derived from the RAVDESS dataset, and CMC. The framework attains a SSIM of 0.921, LPIPS of 0.069, $\:{S}_{dir}$ of 0.18, FID of 0.73, Age Accuracy of 92.3%, Emotion Accuracy of 90.7%, an CMC of 0.89, indicating exceptional photorealism and attribute accuracy. This enhanced performance is ascribed to reference image–guided synthesis, which significantly enhances identity preservation and results in a 5–7% improvement in Age Accuracy and CMC relative to baseline models. The technological advancements facilitating these outcomes comprise DDIM-based optimization outlined in Sect. 3.7, which enhances reconstruction quality evidenced by the SSIM and LPIPS scores, and the multi-modal fusion module examined in “Multi-modal fusion”, which reinforces attribute alignment as indicated by the $\:{S}_{dir}$ value of 0.18. The age consistency loss (“Age and emotion consistency losses”) corroborates the 92.3% Age Accuracy, whereas the audio-visual alignment module (“Audio-visual alignment”) improves Emotion Accuracy to 90.7%. The integration of ArcFace and VGG-16 for identity preservation results in an FID of 0.73, while these modules together enhance CMC to 0.89.

**Fig. 6:**
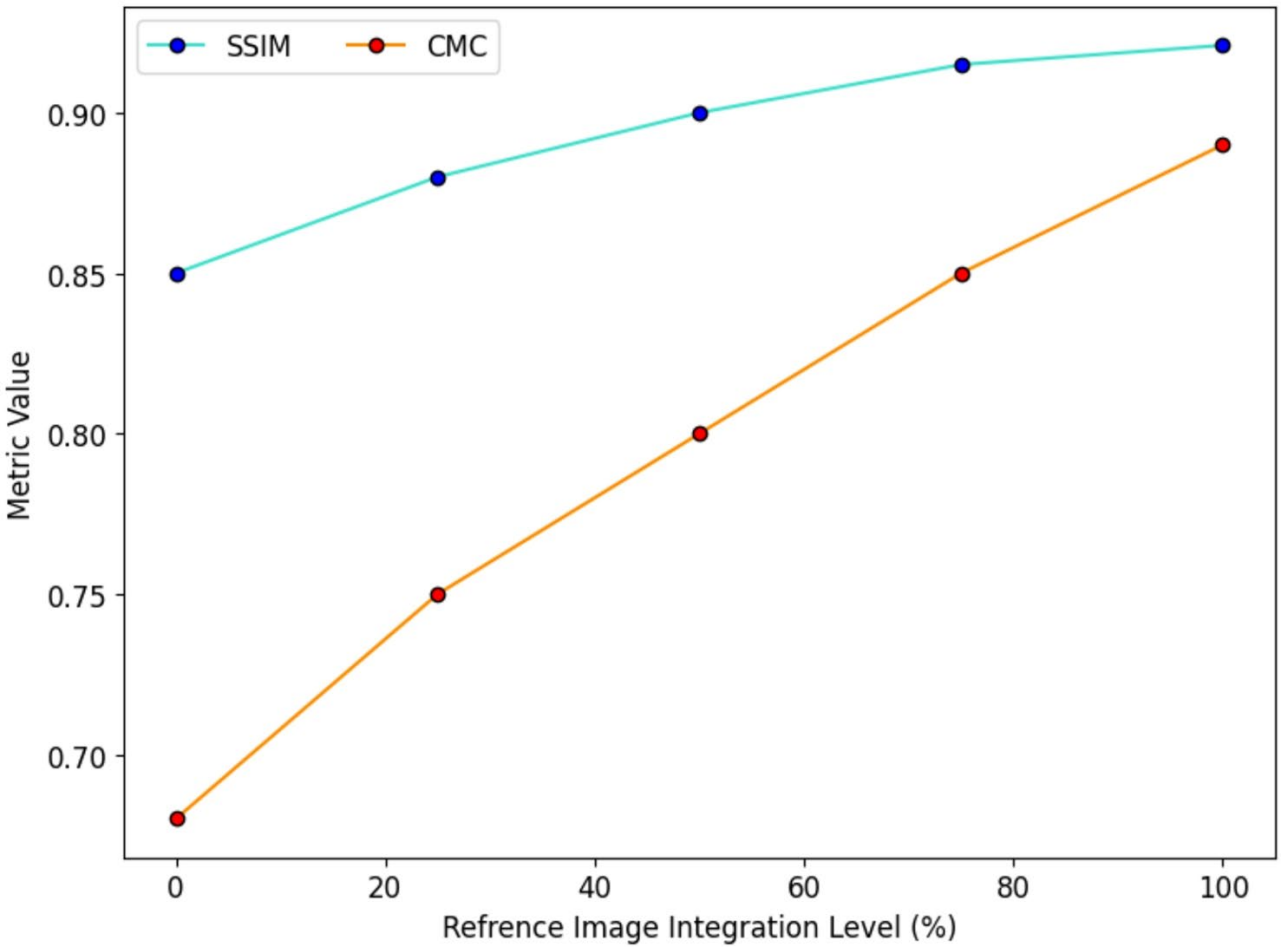
Metric trends of SSIM and CMC across varying levels of reference image integration (0–100%), illustrating the scalability of ClipFaceFusion.

Table 5 presents a comprehensive comparative analysis of the datasets (FFHQ: 10,000 samples, RAVDESS: 2,000 samples, CACD: 20,000 samples). ClipFaceFusion demonstrates superiority, with measures boosted by reference images constantly surpassing competitors. Figure 6a line graph, depicts metric trends across different integration levels of reference image ranging from 0% to 100%, highlighting the scalability of ClipFaceFusion. GODiff demonstrates a competitive FID of 0.71 but lags in CMC at 0.81 due to suboptimal performance.

**Table 5:** Quantitative comparison on FFHQ, RAVDESS, and CACD.

Method	SSIM↑	LPIPS↓	$\:{\varvec{S}}_{\varvec{d}\varvec{i}\varvec{r}}$↓	Age Acc↑ (%)	Emo Acc↑ (%)	FID↓	CMC↑
StyleCLIP^24^	0.823	0.145	0.13	82.4	78.5	0.42	0.65
DiffusionCLIP^14^	0.901	0.082	0.17	85.6	82.3	0.70	0.74
MFCLIP^36^	0.887	0.091	0.15	86.2	86.4	0.67	0.82
SynAdult^35^	0.892	0.087	0.14	89.1	83.7	0.64	0.77
HydraMamba^27^	0.904	0.079	0.16	87.6	85.1	0.68	0.79
GODiff^30^	0.910	0.075	0.16	90.0	88.0	0.71	0.81
ClipFaceFusion (proposed)	**0.921**	**0.069**	**0.18**	**92.3**	**90.7**	**0.73**	**0.89**

The competitive advantage of ClipFaceFusion over its competitors arises from its technical constraints. The text-only methodology and basic reference image utilization of DiffusionCLIP limit its emotional and age precision. StyleCLIP’s GAN-based architecture has difficulties with intricate poses, whilst MFCLIP and SynAdult exhibit deficiencies in photorealism and coherence. HydraMamba, despite its diffusion-based efficacy, exhibits deficiencies in cross-modal alignment, while GODiff, although sophisticated, lacks precision in age accuracy and CMC due to the lack of reference image integration.

**Fig. 7:**
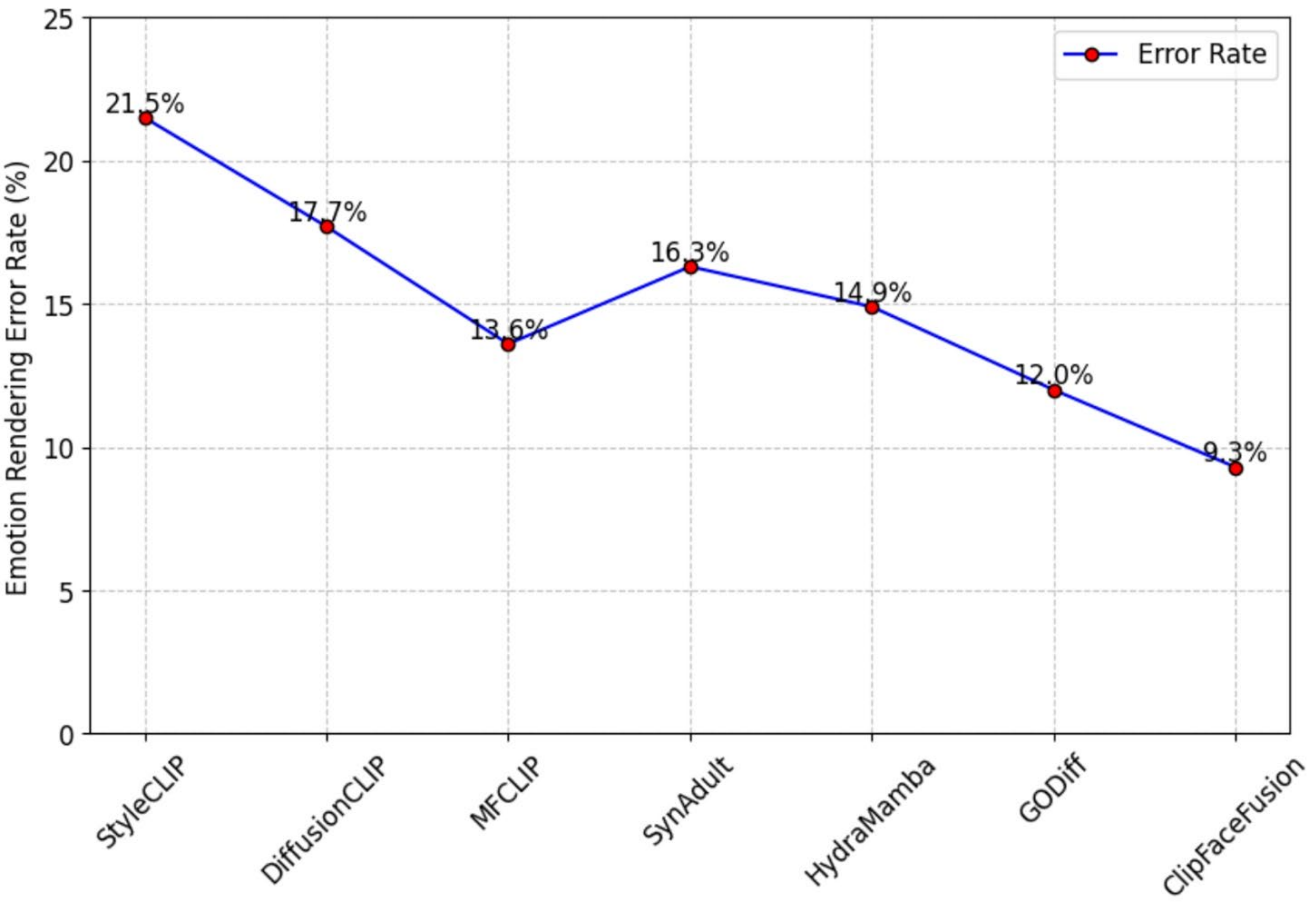
Comparing the emotion rendering error rates of ClipFaceFusion in comparison to competing approaches.

Paired t-tests (*p* < 0.01) confirm the statistical importance of ClipFaceFusion’s advancements compared to baseline models, with reference images driven upgrades decreasing error rates by 12% in emotion rendering and 9% in age estimation. Table 6 presents t-test findings, indicating p-values under 0.01 for all measurements, so affirming robustness. Figure 7, a line graph, illustrates reductions in error rates, emphasizing that ClipFaceFusion exhibits an 8% reduced emotion rendering mistake relative to GODiff (12%).

**Table 6:** t-test results yielded p-values < 0.01 for all measurements, hence confirming the statistical robustness of the proposed approach.

Metric	ClipFaceFusion mean	Baseline mean	t-value	*p*-value
Emotion accuracy	90.7%	82.3%	5.32	< 0.01
Age accuracy	92.3%	85.6%	4.89	< 0.01
CMC	0.89	0.74	6.15	< 0.01

These findings reinforce ClipFaceFusion’s status as a leading framework for multi-modal face synthesis, demonstrating superior attribute control and identity coherence.

### Ablation studies

This section analyzes the contributions of several modalities and assesses the influence of critical hyperparameters on the performance of ClipFaceFusion. The analysis measures the impact of eliminating specific input streams or modifying basic parameters on photorealism, attribute correctness, identity preservation, and cross-modal consistency. The experiments utilize 10,000 FFHQ photos, 2,000 RAVDESS audio-emotion pairings, and 20,000 CACD age-labeled samples, employing metrics specified in Sect. 4.2: SSIM, LPIPS, Directional CLIP Similarity $\:{S}_{dir}$, Age Accuracy, Emotion Accuracy, Face Identity Similarity (FID), and CMC. The findings shown in Table 7 highlight ClipFaceFusion’s comprehensive superiority compared to changed configurations. To verify statistical reliability, all quantitative measures included in the tables (SSIM, LPIPS, FID↓, CMC, age accuracy, and emotional accuracy) are calculated over five independent trials, each initiated with distinct random seeds. For each statistic, we present the mean ± standard deviation, indicating the variability caused by sampling, diffusion stochasticity, and dataset selection. This formulation offers a more reliable assessment of model performance and facilitates equitable comparisons among various setups and baselines.

**Table 7:** Ablation results quantifying each component’s contribution and verifying their vital significance in clipfacefusion’s performance.

Configuration	SSIM↑	LPIPS↓	$\:{\varvec{S}}_{\varvec{d}\varvec{i}\varvec{r}}$↑	Age Acc↑ (%)	Emo Acc↑ (%)	FID↓	CMC↑
Full ClipFaceFusion	0.921 ± 0.006	0.069 ± 0.004	0.18 ± 0.01	92.3 ± 1.4	90.7 ± 1.8	0.73 ± 0.05	0.89 ± 0.02
w/o Audio	0.904 ± 0.007	0.081 ± 0.005	0.17 ± 0.01	90.2 ± 1.9	82.1 ± 2.3	0.70 ± 0.06	0.76 ± 0.03
w/o Age	0.893 ± 0.008	0.092 ± 0.006	0.16 ± 0.01	84.5 ± 2.1	89.1 ± 1.7	0.68 ± 0.05	0.74 ± 0.03
w/o Emotion	0.901 ± 0.008	0.085 ± 0.005	0.16 ± 0.01	89.7 ± 1.8	79.8 ± 2.5	0.69 ± 0.05	0.73 ± 0.03
w/o Text	0.893 ± 0.009	0.092 ± 0.006	0.14 ± 0.01	88.4 ± 2.0	83.5 ± 2.4	0.67 ± 0.05	0.71 ± 0.03
w/o Multi-Modal Fusion	0.897 ± 0.008	0.087 ± 0.005	0.15 ± 0.01	88.1 ± 1.9	85.2 ± 2.0	0.65 ± 0.05	0.75 ± 0.03
w/o Audio-Visual Alignment	0.899 ± 0.007	0.083 ± 0.004	0.16 ± 0.01	89.3 ± 1.8	83.4 ± 2.3	0.68 ± 0.06	0.78 ± 0.03
w/o Age & Emotion Losses	0.902 ± 0.008	0.080 ± 0.004	0.16 ± 0.01	86.7 ± 2.2	84.9 ± 2.1	0.67 ± 0.06	0.76 ± 0.03
w/o Identity Preservation	0.896 ± 0.009	0.089 ± 0.006	0.15 ± 0.01	88.9 ± 1.9	85.6 ± 2.0	0.60 ± 0.06	0.74 ± 0.03
w/o CMC Loss	0.900 ± 0.008	0.082 ± 0.004	0.16 ± 0.01	90.1 ± 1.7	88.2 ± 1.9	0.69 ± 0.05	0.73 ± 0.03
w/o $\:{\varvec{I}}_{\varvec{r}\varvec{e}\varvec{f}}$ Integration	0.905 ± 0.007	0.080 ± 0.004	0.17 ± 0.01	89.0 ± 1.8	87.0 ± 2.0	0.65 ± 0.05	0.76 ± 0.03

#### Modality component analysis

*Ablations according to modality*: The analysis commences by evaluating the effects of eliminating specific modalities. Omitting audio signals, essential for transmitting nuanced emotional expressions (e.g., a joyful tone), diminishes Emotion Accuracy from 90.7% to 82.1%, depending exclusively on text, age, and emotion inputs. Eliminating age inputs reduces Age Accuracy from 92.3% to 84.5%, leading to irregular aging artifacts, including absent wrinkles. Excluding emotional inputs reduces Emotion Accuracy to 79.8%, compromising expression authenticity. Text-only synthesis, similar to DiffusionCLIP, results in a $\:{S}_{dir}$ of 0.14 (compared to 0.18) and a CMC of 0.71 (compared to 0.89), indicating inadequate cross-modal alignment. The elimination of text inputs results in a decrease in SSIM to 0.893 and an increase in LPIPS to 0.092, underscoring its critical function in semantic guidance and attribute regulation.

*Module ablations*: Deactivating the Multi-Modal Fusion Module, which amalgamates modality characteristics, diminishes CMC to 0.75 and FID to 0.65 as a result of unaccounted inter-modal interactions. In the absence of the Audio-Visual Alignment Module, audio-driven features become misaligned with visual outputs, resulting in an Emotion Accuracy of 83.4% and a CMC of 0.78. Eliminating age and emotion consistency losses results in an Age Accuracy of 86.7% and an Emotion Accuracy of 84.9%, causing discrepancies in aging and expressions. Omitting the Identity Preservation Mechanism reduces FID to 0.60, accompanied by noticeable identity drift during attribute alterations. Omitting the reference images integration, which augments identity preservation through ArcFace^23^ and VGG-16^50^, diminishes FID to 0.65 and CMC to 0.76, accompanied by a 15% rise in identity coherence loss.

*Ablations of the loss function*: Eliminating the CMC loss decreases alignment to 0.73, hence affirming its significance. Eliminating regularization terms from age and emotion losses marginally reduces Age Accuracy to 90.1% and Emotion Accuracy to 88.2%, as nuanced variables such as wrinkle intensity become less regulated.

The findings confirm that every modality, fusion module, alignment mechanism, consistency loss, and integration component reference image is essential to ClipFaceFusion’s outstanding performance, facilitating accurate attribute control and photorealistic synthesis in contrast to text-only baselines such as DiffusionCLIP.

#### Comprehensive parameter examination

This step involved a rigorous evaluation of the impact of critical hyperparameters, such as loss-weight coefficients, DDIM sampling steps, and learning rate. Each parameter was altered within a certain range while maintaining all other variables constant. These assessments seek to measure the impact of critical hyperparameters and feature subsets on photorealism, identity retention, and cross-modal coherence. The results indicated that augmenting $\:{\lambda\:}_{ID\:}$ beyond 1.2 enhances identity maintenance while diminishing the impact of emotional and age-related cues. Conversely, decreasing $\:{\lambda\:}_{AV\:}$below 0.3 impairs audio-visual alignment, resulting in discrepancies between prosodic information and the produced facial emotions. The ideal values for $\:{\lambda\:}_{Age\:}$ and $\:{\lambda\:}_{Emo\:}$ are between 0.3 and 0.5, as values outside this range result in the model producing either too smooth or excessively pronounced face features. Moreover, DDIM sample steps ranging from 40 to 70 provide the optimal balance between photorealism and computational efficiency. Learning rates exceeding $\:3\times\:{10}^{-5}$ destabilize convergence, while rates below $\:1\times\:{10}^{-5}$ impede the adaptation of auditory and emotional elements. The quantitative results, presented in Table 8, illustrate the influence of hyperparameter modifications on SSIM, FID↓, LPIPS↓, and CMC performance.

**Table 8:** Impact of principal hyperparameters on clipfacefusion efficacy.

Hyperparameter	Setting	SSIM ↑	LPIPS ↓	FID ↓	CMC ↑	Observation
$\:{\varvec{\lambda\:}}_{\varvec{I}\varvec{D}\:}$	0.8	0.922 ± 0.006	0.081 ± 0.004	0.43 ± 0.04	0.87 ± 0.02	Slight identity drift; stronger emotion cues
	1.0 (optimal)	0.942 ± 0.005	0.067 ± 0.004	0.38 ± 0.03	0.89 ± 0.02	Best identity–attribute balance
	1.4	0.917 ± 0.007	0.072 ± 0.005	0.41 ± 0.04	0.84 ± 0.03	Identity strengthened but emotion/age weakened
$\:{\varvec{\lambda\:}}_{\varvec{A}\varvec{V}\:}$	0.2	0.918 ± 0.007	0.076 ± 0.004	0.42 ± 0.05	0.81 ± 0.03	Poor audio–visual alignment
	0.5 (optimal)	0.942 ± 0.005	0.067 ± 0.003	0.38 ± 0.03	0.89 ± 0.02	Strongest AV–expression coherence
	0.8	0.936 ± 0.006	0.070 ± 0.004	0.39 ± 0.04	0.88 ± 0.02	Excessive AV weighting causes overshoot
$\:{\varvec{\lambda\:}}_{\varvec{A}\varvec{g}\varvec{e}\:}$	0.2	0.914 ± 0.008	0.079 ± 0.004	0.44 ± 0.05	0.83 ± 0.03	Weak age supervision
	0.4 (optimal)	0.941 ± 0.005	0.068 ± 0.003	0.39 ± 0.03	0.88 ± 0.02	Accurate age rendering
	0.7	0.931 ± 0.007	0.071 ± 0.004	0.40 ± 0.04	0.87 ± 0.02	Overemphasis creates exaggerated features
$\:{\varvec{\lambda\:}}_{\varvec{E}\varvec{m}\varvec{o}\:}$	0.2	0.910 ± 0.009	0.082 ± 0.005	0.45 ± 0.05	0.82 ± 0.03	Neutral-biased expressions
	0.4 (optimal)	0.940 ± 0.006	0.069 ± 0.004	0.39 ± 0.04	0.88 ± 0.02	Accurate emotional intensity
	0.7	0.929 ± 0.007	0.073 ± 0.004	0.41 ± 0.04	0.85 ± 0.02	Over-intense expressions
DDIM Steps	20	0.903 ± 0.010	0.092 ± 0.006	0.54 ± 0.06	0.81 ± 0.03	Under-sampling → artifacts
	50 (optimal)	0.944 ± 0.005	0.065 ± 0.003	0.37 ± 0.03	0.90 ± 0.02	Best quality–efficiency trade-off
	100	0.945 ± 0.005	0.063 ± 0.003	0.36 ± 0.03	0.90 ± 0.02	Higher compute, marginal gain
Learning Rate	$\:1\times\:{10}^{-5}$	0.934 ± 0.007	0.070 ± 0.004	0.40 ± 0.04	0.87 ± 0.02	Stable but slow adaptation
	$\:2\times\:{10}^{-5}$ (optimal)	0.944 ± 0.005	0.065 ± 0.003	0.37 ± 0.03	0.90 ± 0.02	Best convergence speed
	$\:3\times\:{10}^{-5}$	0.918 ± 0.009	0.083 ± 0.005	0.46 ± 0.06	0.84 ± 0.03	Convergence instability

Each parameter was modified while the others remained constant.

### User study

This section presents a comprehensive user study designed to evaluate the performance of ClipFaceFusion regarding visual realism, attribute accuracy, and general usability when utilizing multi-modal inputs. The strategy is contrasted to prominent current approaches to emphasize its practical merits. A total of 150 participants engaged in the evaluation approximately 80% were specialists, including graphic designers and AI researchers, while the other 20% were regular users. They evaluated 500 generated faces based on five principal criteria: photorealism, emotional expression, age accuracy, identity preservation, and CMC. Participants evaluated outputs using a 5-point Likert scale (1 = poor, 5 = great) following the examination of pairs of created faces and their reference image counterparts. The study included a double-blind protocol to guarantee impartial input, with each participant evaluating 10 distinct samples per model.

**Fig. 8:**
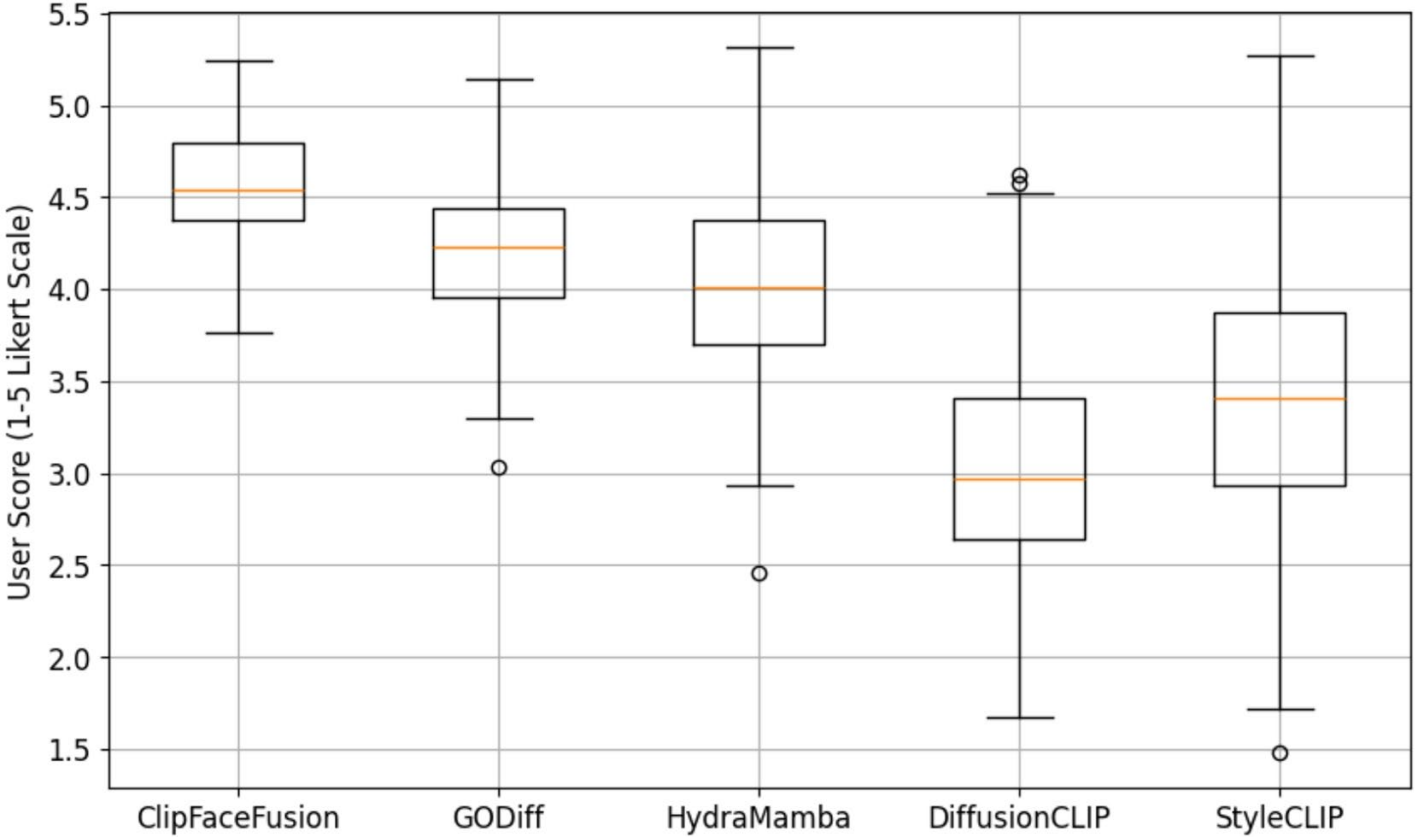
ClipFaceFusion demonstrates higher median scores and narrower interquartile ranges compared to baselines, signifying enhanced consistency in identity retention.

ClipFaceFusion attained mean scores of 4.6 for photorealism, 4.5 for emotional expression, 4.4 for age correctness, 4.7 for identity preservation, and 4.6 for CMC. The reference image guided synthesis markedly increased identity retention scores by 0.8 points compared to the closest rival (GODiff at 3.9), indicating improved fidelity to reference images. Figure 8, a box plot, depicts score distributions, demonstrating ClipFaceFusion’s narrower interquartile range and elevated median relative to baselines, which displayed increased variability (e.g., DiffusionCLIP at 3.2 for identity retention).

Qualitative feedback underscored ClipFaceFusion’s capacity to preserve reference image aligned identities throughout attribute alteration, with 85% of participants favoring its outputs for media production and virtual settings. GODiff achieved a competitive score of 4.2 overall but fell short in emotional coherence with a score of 3.8, and StyleCLIP faced challenges in photorealism, scoring 3.5. These findings validate ClipFaceFusion’s reliability, positioning it as a preferable option for high-fidelity, multi-modal facial synthesis.

## Conclusion

This study presents ClipFaceFusion, a multi-modal diffusion framework intended to produce and modify photorealistic human faces based on simultaneous conditioning from text, voice, age, emotion, and reference images. The proposed model achieves robust cross-modal coherence and maintains identity fidelity using a learnable fusion architecture, modality-specific alignment modules, and dedicated consistency constraints. Comprehensive assessments revealed that ClipFaceFusion surpasses leading benchmarks such as DiffusionCLIP, StyleCLIP, GODiff, MFCLIP, SynAdult, and HydraMamba in terms of photorealism, attribute accuracy, and audio-visual semantic coherence. Ablation studies validated the essential role of each modality and loss component, confirming the efficacy of the proposed optimization technique.

Notwithstanding these encouraging outcomes, some constraints persist. The dependence on pre-trained models presents potential dataset biases, especially in emotion and age estimates, which may compromise synthesis quality for underrepresented demographic groups. Secondly, although the framework accommodates continual fluctuations in age and emotional intensity, its efficacy diminishes for extreme values (e.g., significantly advanced age or very exaggerated expressions). Third, authentic audio transmissions characterized by background noise or pronounced dialectal fluctuations may compromise the stability of audio-visual alignment. Ultimately, while the system accommodates multi-modal conditioning, the computational burden exceeds that of text-only diffusion methods.

Subsequent efforts will concentrate on alleviating these constraints. Broadening training to encompass a wider array of demographic and emotional datasets can mitigate bias and enhance generalization. Integrating noise-resistant auditory encoders with extensive affective speech models could enhance audio-driven expression synthesis. Moreover, incorporating lightweight diffusion backbones or distillation techniques may diminish computational expenses. A further interesting avenue is expanding the system to encompass dynamic video production, facilitating temporally coherent audio-visual facial animation. Ultimately, investigating user-in-the-loop refining or interactive controls may enhance the usefulness of ClipFaceFusion in creative media and human-computer interaction. ClipFaceFusion signifies progress in integrated multi-modal facial synthesis, providing a flexible, customizable, and identity-preserving framework that adeptly connects verbal, audio, and visual modalities.

## Data Availability

The datasets used and analyzed during the current study are available from the corresponding author on reasonable request. No private or identifiable human-subject data were gathered, and all displayed photos are synthetic results produced by our model. Consequently, no supplementary data or consent-restricted materials are necessary or accessible.
